# Supplementary figures and images for: Computational Screening of Tip and Stalk Cell Behavior Proposes a Role for Apelin Signaling in Sprout Progression
Source: PLoS One. 2016 Nov 9;11(11):e0159478. doi: 10.1371/journal.pone.0159478 (PMC5102492; doi:10.1371/journal.pone.0159478)

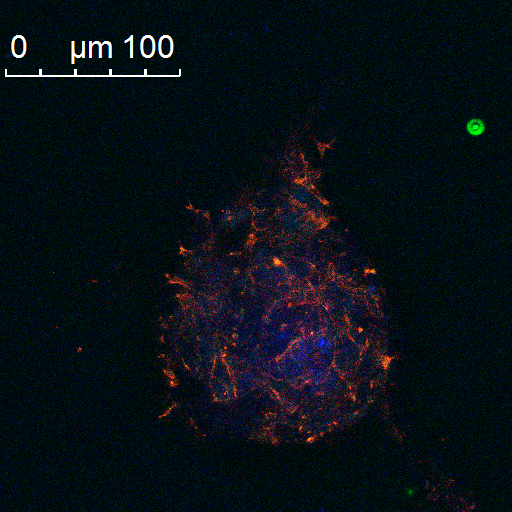

Supplement: S2 Dataset — Images in TIFF format. The measurements (performed using ImageJ) are given in file “cellareas.xlsx”. (ZIP) [file pone.0159478.s007.zip › sproutende ECs/Series007_z14.tif]

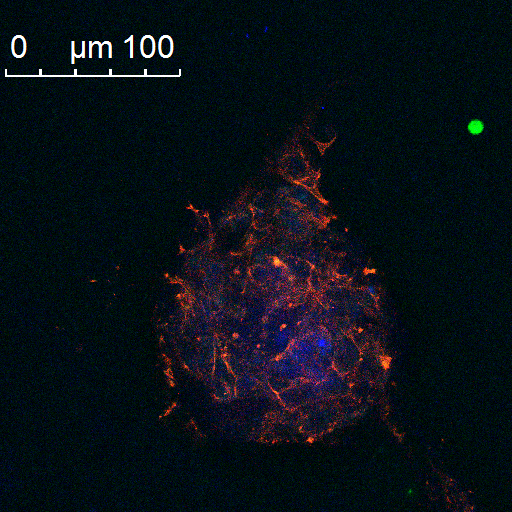

Supplement: S2 Dataset — Images in TIFF format. The measurements (performed using ImageJ) are given in file “cellareas.xlsx”. (ZIP) [file pone.0159478.s007.zip › sproutende ECs/Series007_z15.tif]

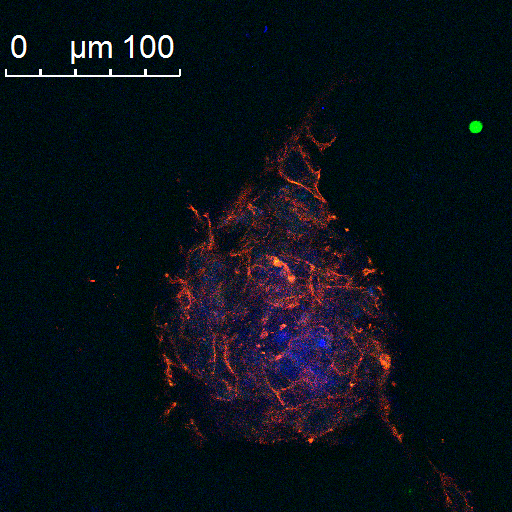

Supplement: S2 Dataset — Images in TIFF format. The measurements (performed using ImageJ) are given in file “cellareas.xlsx”. (ZIP) [file pone.0159478.s007.zip › sproutende ECs/Series007_z16.tif]

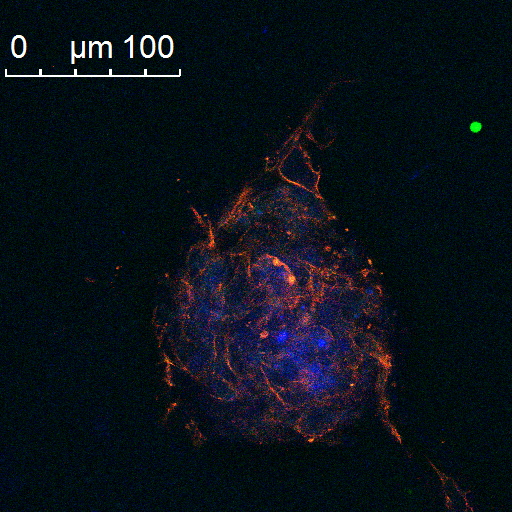

Supplement: S2 Dataset — Images in TIFF format. The measurements (performed using ImageJ) are given in file “cellareas.xlsx”. (ZIP) [file pone.0159478.s007.zip › sproutende ECs/Series007_z17.tif]

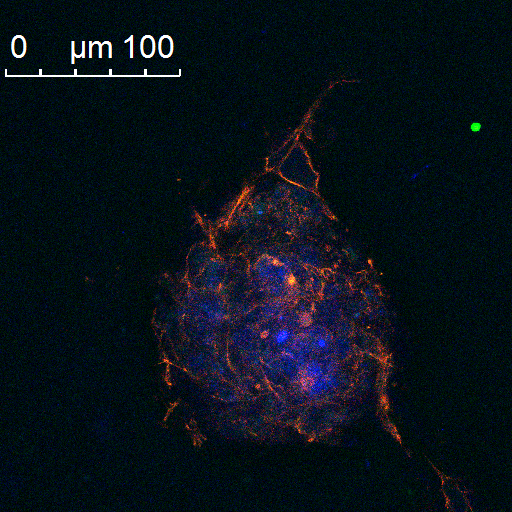

Supplement: S2 Dataset — Images in TIFF format. The measurements (performed using ImageJ) are given in file “cellareas.xlsx”. (ZIP) [file pone.0159478.s007.zip › sproutende ECs/Series007_z18.tif]

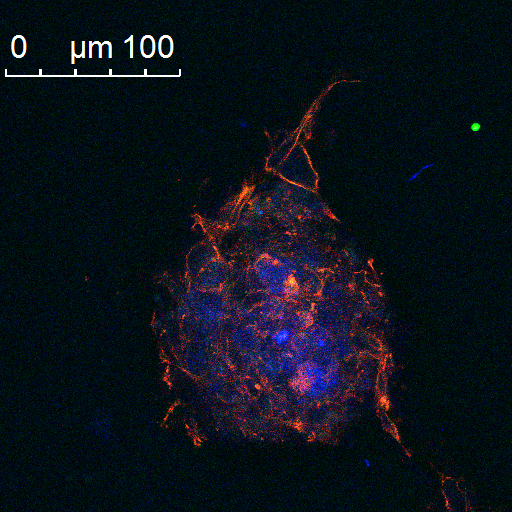

Supplement: S2 Dataset — Images in TIFF format. The measurements (performed using ImageJ) are given in file “cellareas.xlsx”. (ZIP) [file pone.0159478.s007.zip › sproutende ECs/Series007_z19.tif]

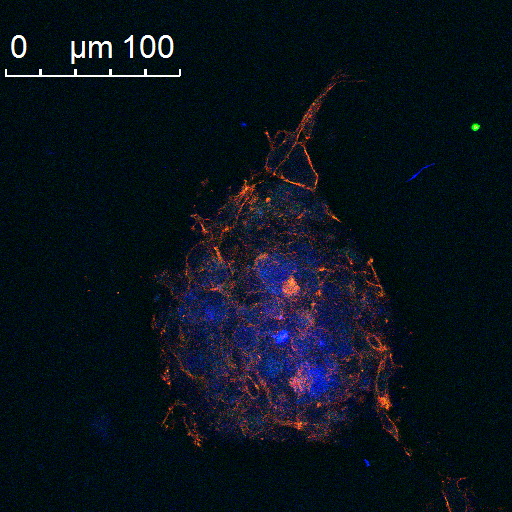

Supplement: S2 Dataset — Images in TIFF format. The measurements (performed using ImageJ) are given in file “cellareas.xlsx”. (ZIP) [file pone.0159478.s007.zip › sproutende ECs/Series007_z20.tif]

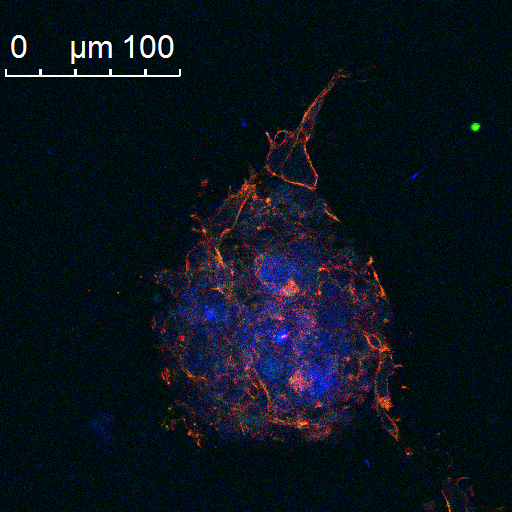

Supplement: S2 Dataset — Images in TIFF format. The measurements (performed using ImageJ) are given in file “cellareas.xlsx”. (ZIP) [file pone.0159478.s007.zip › sproutende ECs/Series007_z21.tif]

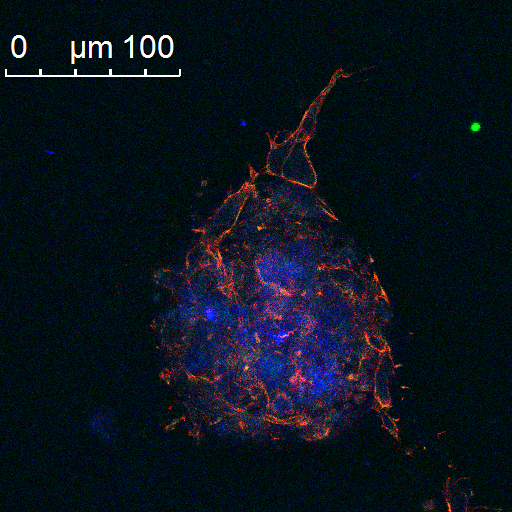

Supplement: S2 Dataset — Images in TIFF format. The measurements (performed using ImageJ) are given in file “cellareas.xlsx”. (ZIP) [file pone.0159478.s007.zip › sproutende ECs/Series007_z22.tif]

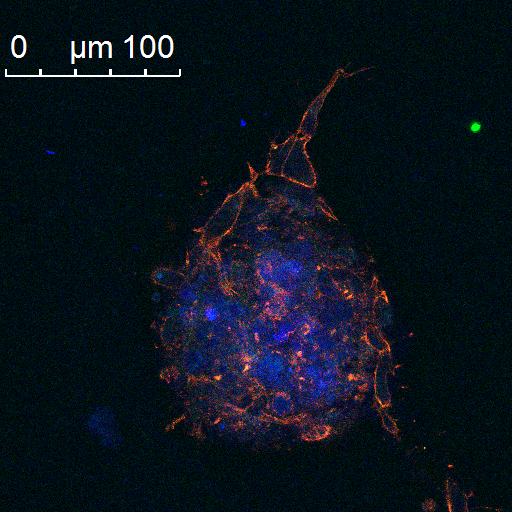

Supplement: S2 Dataset — Images in TIFF format. The measurements (performed using ImageJ) are given in file “cellareas.xlsx”. (ZIP) [file pone.0159478.s007.zip › sproutende ECs/Series007_z23.tif]

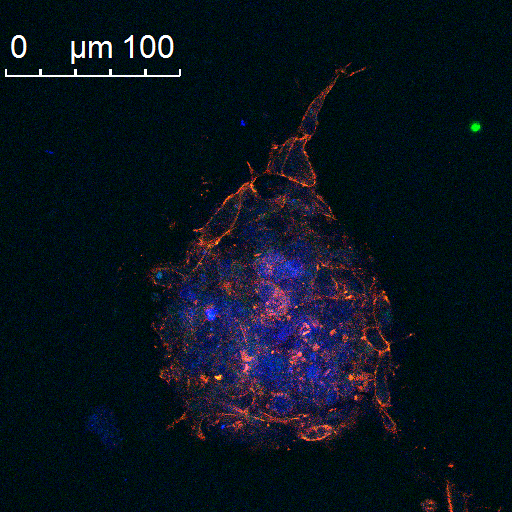

Supplement: S2 Dataset — Images in TIFF format. The measurements (performed using ImageJ) are given in file “cellareas.xlsx”. (ZIP) [file pone.0159478.s007.zip › sproutende ECs/Series007_z24.tif]

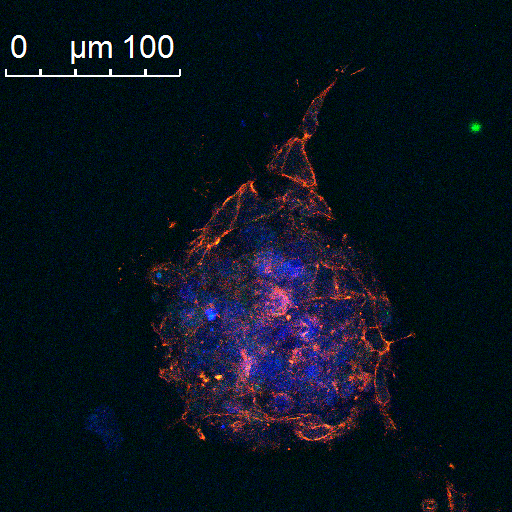

Supplement: S2 Dataset — Images in TIFF format. The measurements (performed using ImageJ) are given in file “cellareas.xlsx”. (ZIP) [file pone.0159478.s007.zip › sproutende ECs/Series007_z25.tif]

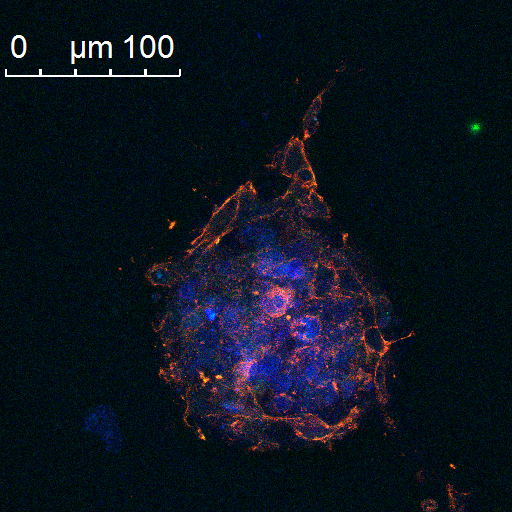

Supplement: S2 Dataset — Images in TIFF format. The measurements (performed using ImageJ) are given in file “cellareas.xlsx”. (ZIP) [file pone.0159478.s007.zip › sproutende ECs/Series007_z26.tif]

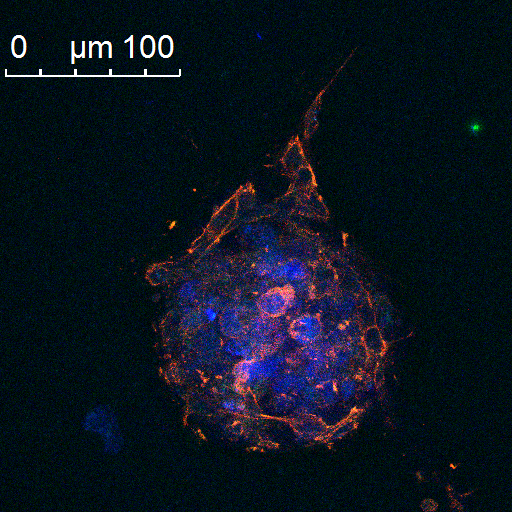

Supplement: S2 Dataset — Images in TIFF format. The measurements (performed using ImageJ) are given in file “cellareas.xlsx”. (ZIP) [file pone.0159478.s007.zip › sproutende ECs/Series007_z27.tif]

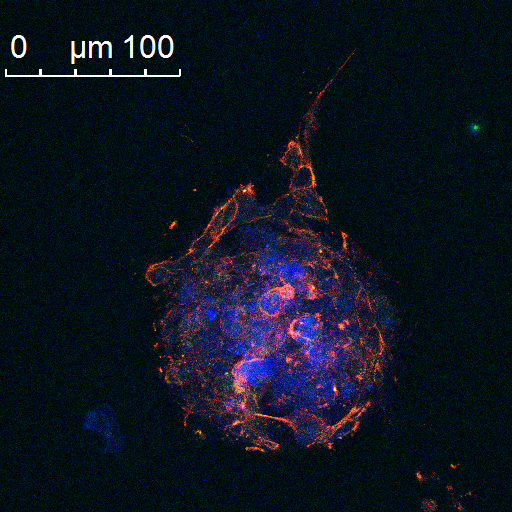

Supplement: S2 Dataset — Images in TIFF format. The measurements (performed using ImageJ) are given in file “cellareas.xlsx”. (ZIP) [file pone.0159478.s007.zip › sproutende ECs/Series007_z28.tif]

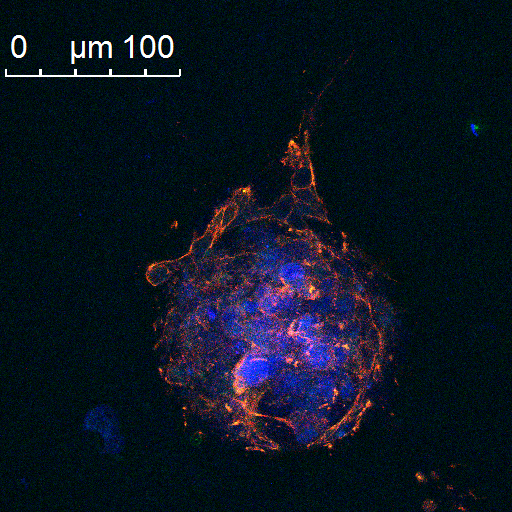

Supplement: S2 Dataset — Images in TIFF format. The measurements (performed using ImageJ) are given in file “cellareas.xlsx”. (ZIP) [file pone.0159478.s007.zip › sproutende ECs/Series007_z29.tif]

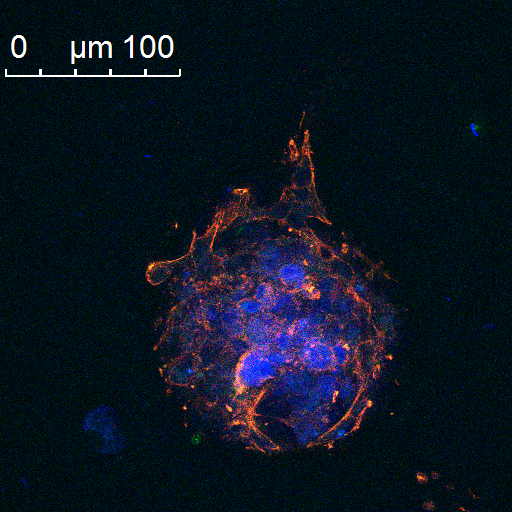

Supplement: S2 Dataset — Images in TIFF format. The measurements (performed using ImageJ) are given in file “cellareas.xlsx”. (ZIP) [file pone.0159478.s007.zip › sproutende ECs/Series007_z30.tif]

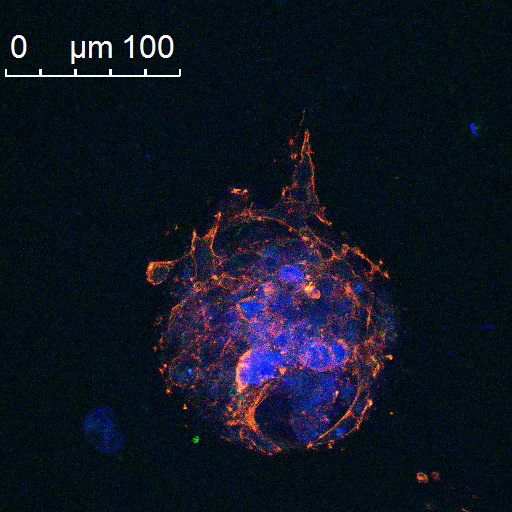

Supplement: S2 Dataset — Images in TIFF format. The measurements (performed using ImageJ) are given in file “cellareas.xlsx”. (ZIP) [file pone.0159478.s007.zip › sproutende ECs/Series007_z31.tif]

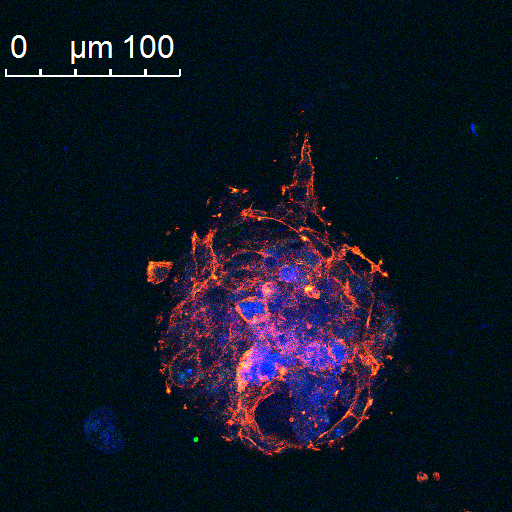

Supplement: S2 Dataset — Images in TIFF format. The measurements (performed using ImageJ) are given in file “cellareas.xlsx”. (ZIP) [file pone.0159478.s007.zip › sproutende ECs/Series007_z32.tif]

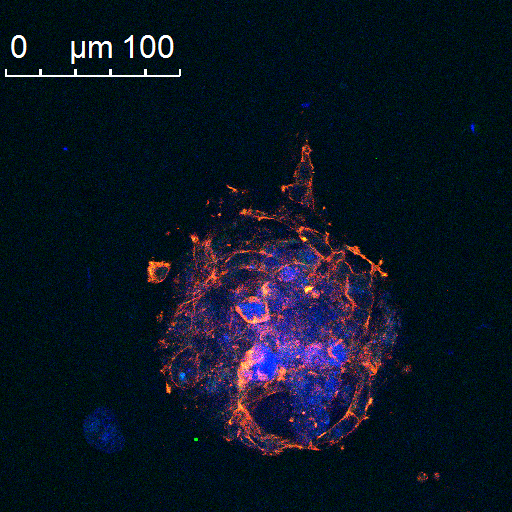

Supplement: S2 Dataset — Images in TIFF format. The measurements (performed using ImageJ) are given in file “cellareas.xlsx”. (ZIP) [file pone.0159478.s007.zip › sproutende ECs/Series007_z33.tif]

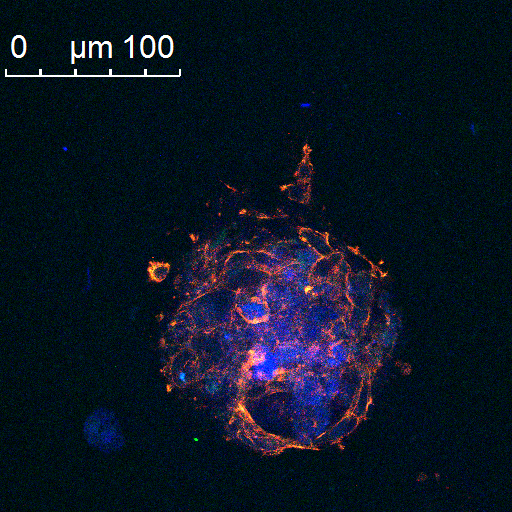

Supplement: S2 Dataset — Images in TIFF format. The measurements (performed using ImageJ) are given in file “cellareas.xlsx”. (ZIP) [file pone.0159478.s007.zip › sproutende ECs/Series007_z34.tif]

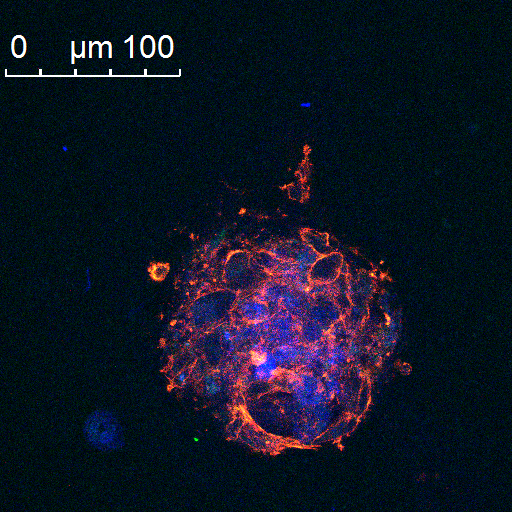

Supplement: S2 Dataset — Images in TIFF format. The measurements (performed using ImageJ) are given in file “cellareas.xlsx”. (ZIP) [file pone.0159478.s007.zip › sproutende ECs/Series007_z35.tif]

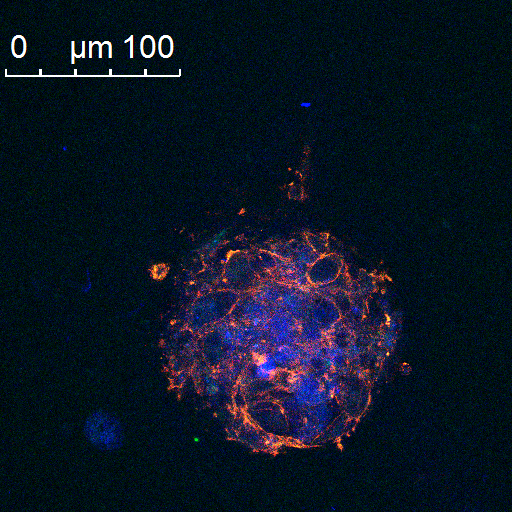

Supplement: S2 Dataset — Images in TIFF format. The measurements (performed using ImageJ) are given in file “cellareas.xlsx”. (ZIP) [file pone.0159478.s007.zip › sproutende ECs/Series007_z36.tif]

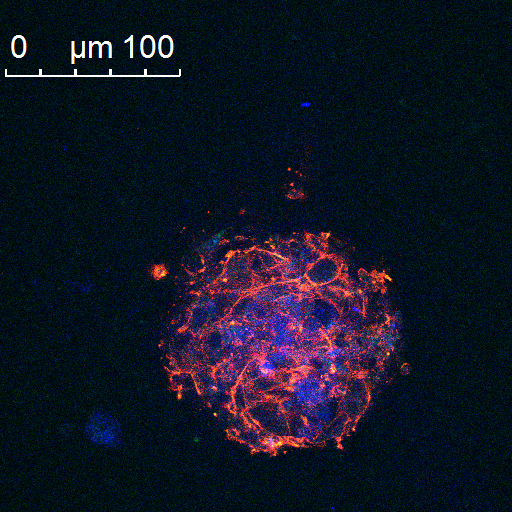

Supplement: S2 Dataset — Images in TIFF format. The measurements (performed using ImageJ) are given in file “cellareas.xlsx”. (ZIP) [file pone.0159478.s007.zip › sproutende ECs/Series007_z37.tif]

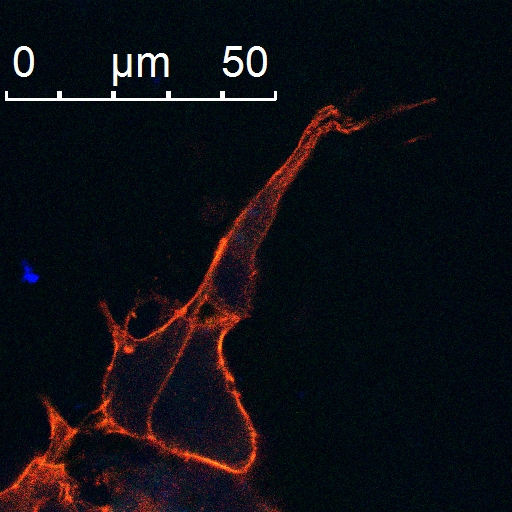

Supplement: S2 Dataset — Images in TIFF format. The measurements (performed using ImageJ) are given in file “cellareas.xlsx”. (ZIP) [file pone.0159478.s007.zip › sproutende ECs/Series010_z0.jpg]

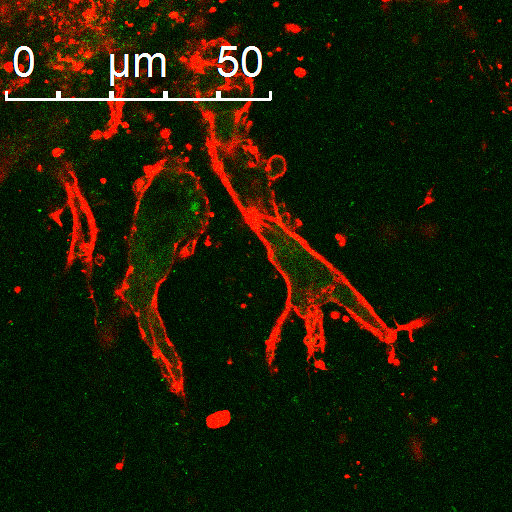

Supplement: S2 Dataset — Images in TIFF format. The measurements (performed using ImageJ) are given in file “cellareas.xlsx”. (ZIP) [file pone.0159478.s007.zip › sproutende ECs/Series030_z0.tif]

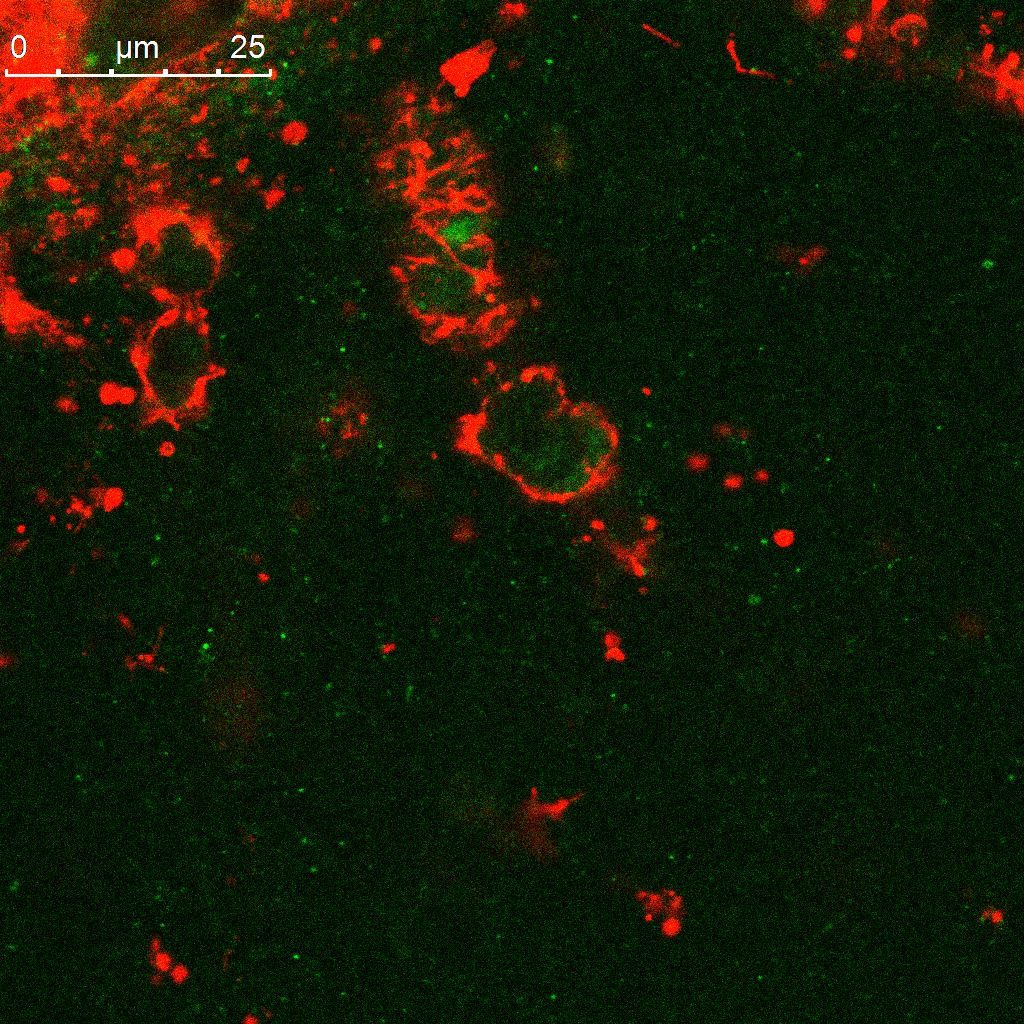

Supplement: S2 Dataset — Images in TIFF format. The measurements (performed using ImageJ) are given in file “cellareas.xlsx”. (ZIP) [file pone.0159478.s007.zip › sproutende ECs/Series035_z08.tif]

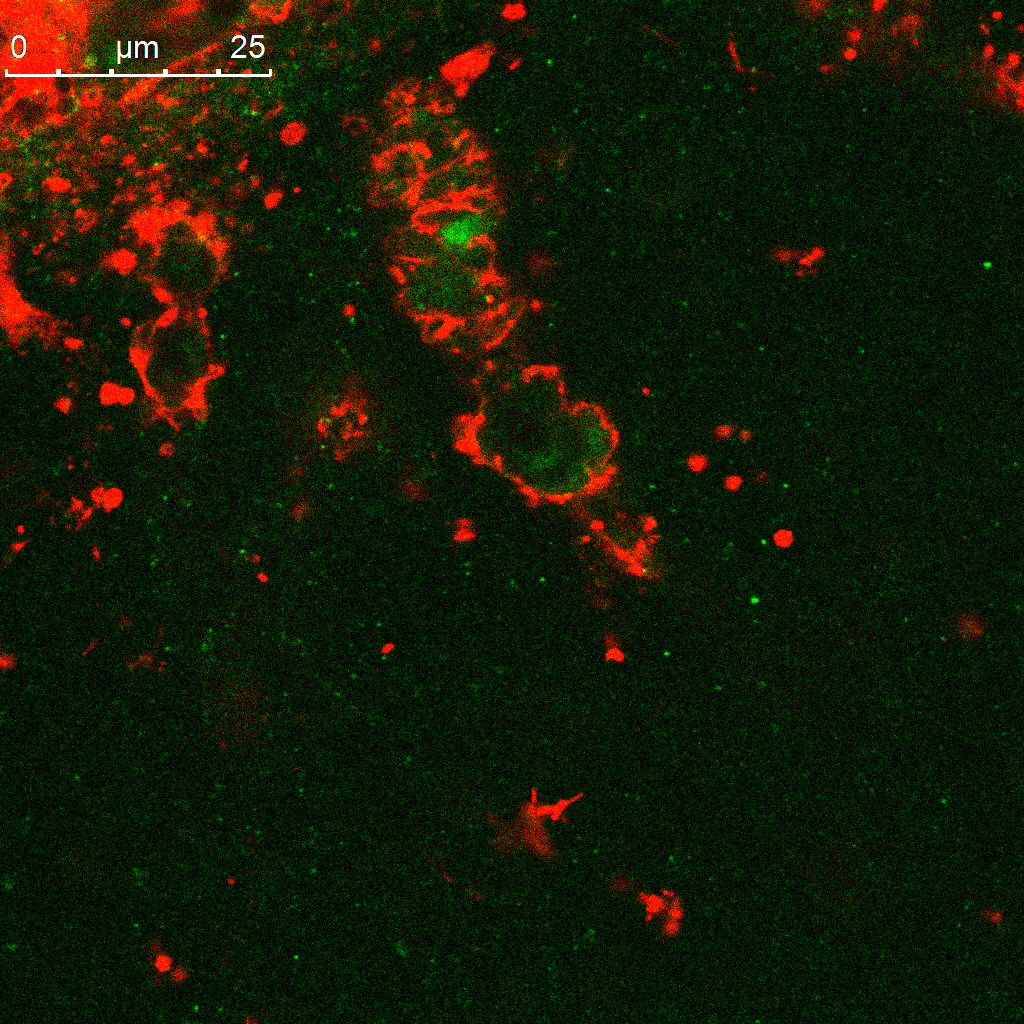

Supplement: S2 Dataset — Images in TIFF format. The measurements (performed using ImageJ) are given in file “cellareas.xlsx”. (ZIP) [file pone.0159478.s007.zip › sproutende ECs/Series035_z09.tif]

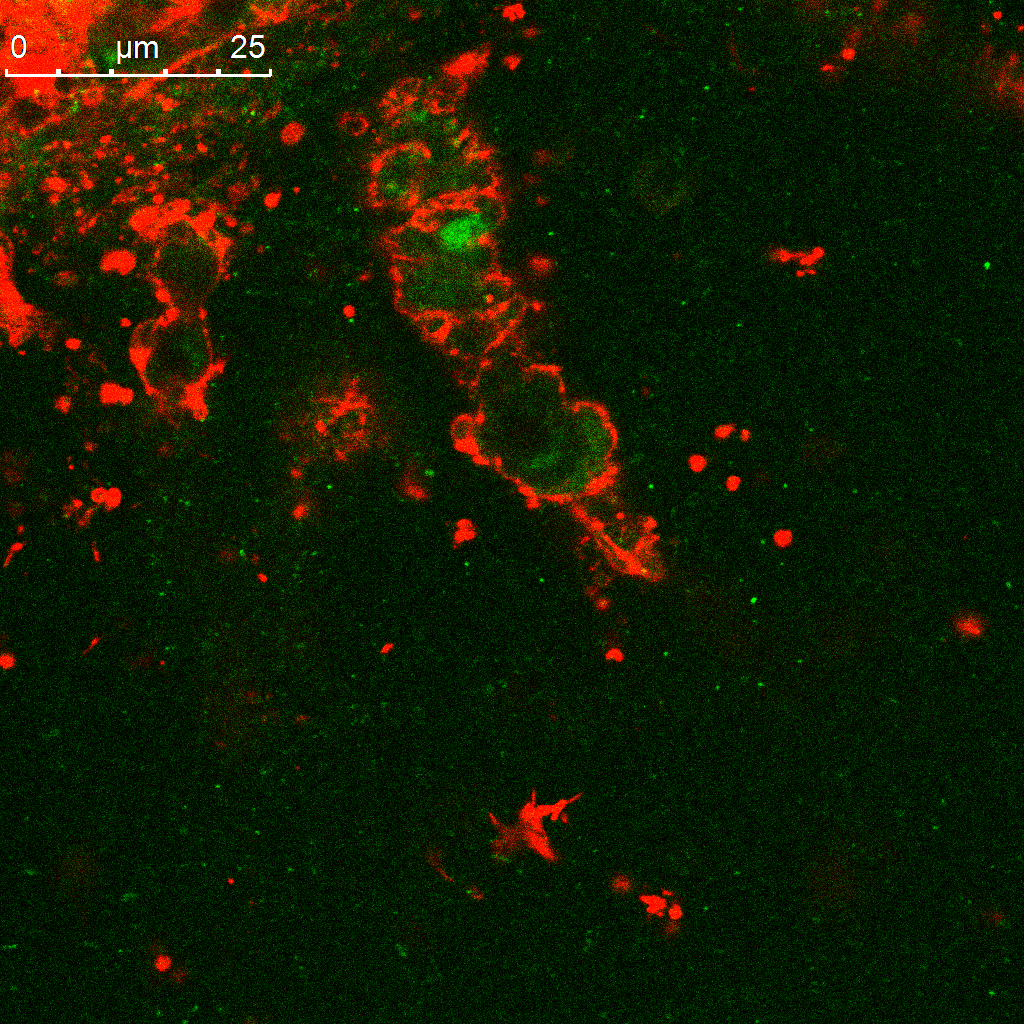

Supplement: S2 Dataset — Images in TIFF format. The measurements (performed using ImageJ) are given in file “cellareas.xlsx”. (ZIP) [file pone.0159478.s007.zip › sproutende ECs/Series035_z10.tif]

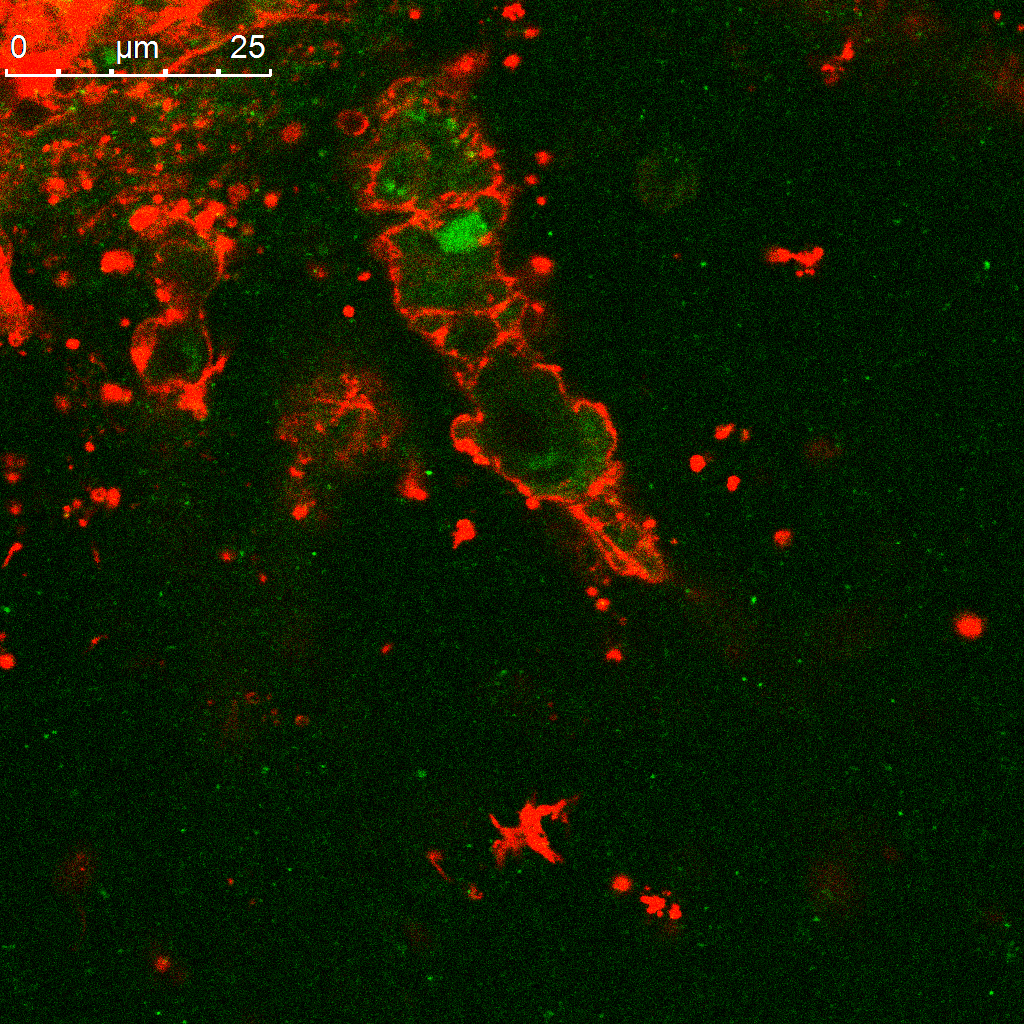

Supplement: S2 Dataset — Images in TIFF format. The measurements (performed using ImageJ) are given in file “cellareas.xlsx”. (ZIP) [file pone.0159478.s007.zip › sproutende ECs/Series035_z11.tif]

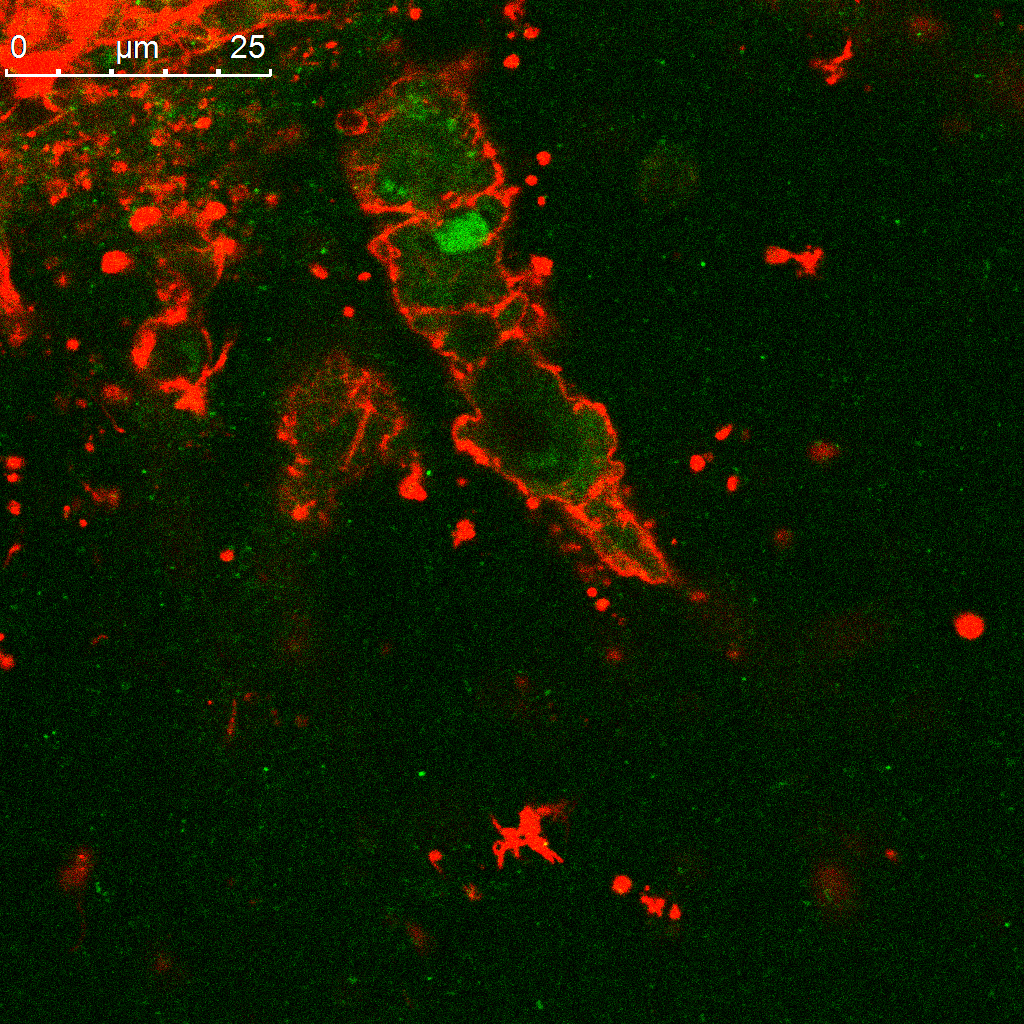

Supplement: S2 Dataset — Images in TIFF format. The measurements (performed using ImageJ) are given in file “cellareas.xlsx”. (ZIP) [file pone.0159478.s007.zip › sproutende ECs/Series035_z12.tif]

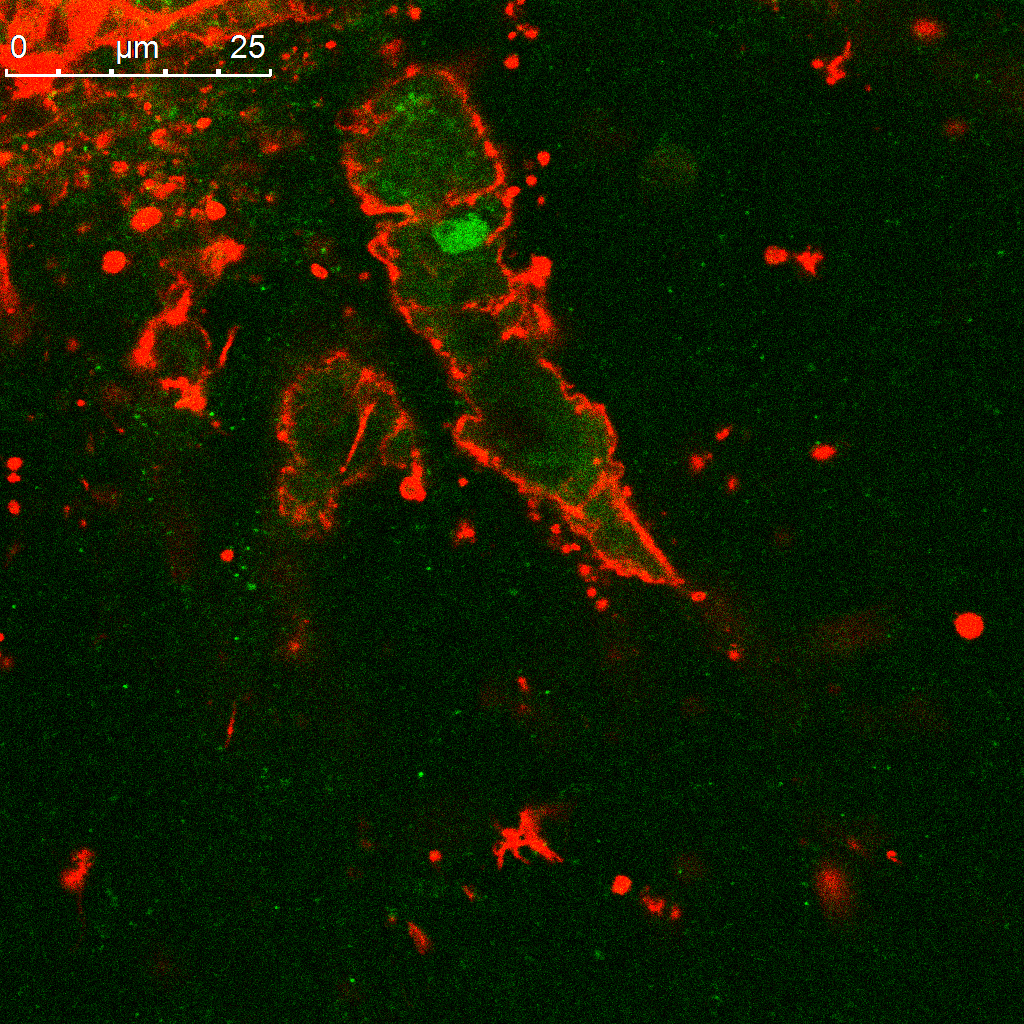

Supplement: S2 Dataset — Images in TIFF format. The measurements (performed using ImageJ) are given in file “cellareas.xlsx”. (ZIP) [file pone.0159478.s007.zip › sproutende ECs/Series035_z13.tif]

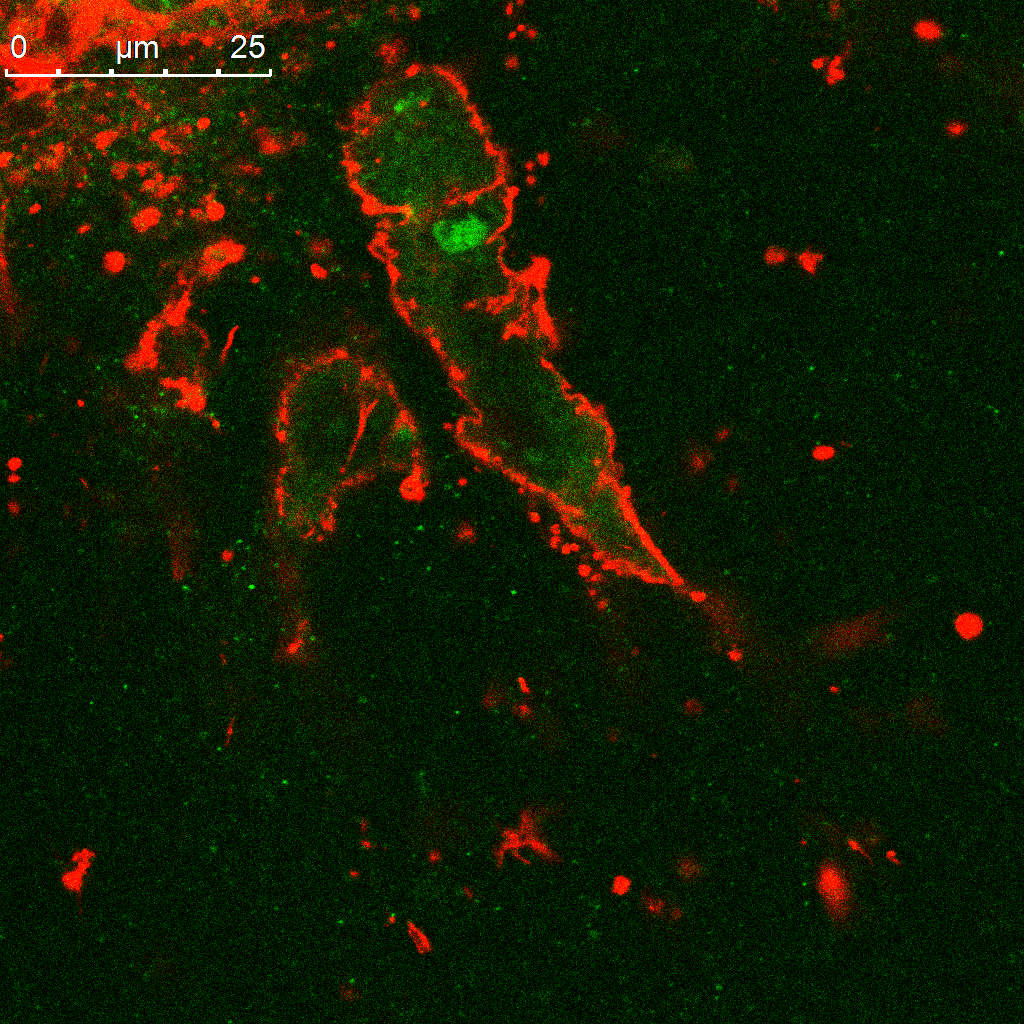

Supplement: S2 Dataset — Images in TIFF format. The measurements (performed using ImageJ) are given in file “cellareas.xlsx”. (ZIP) [file pone.0159478.s007.zip › sproutende ECs/Series035_z14.tif]

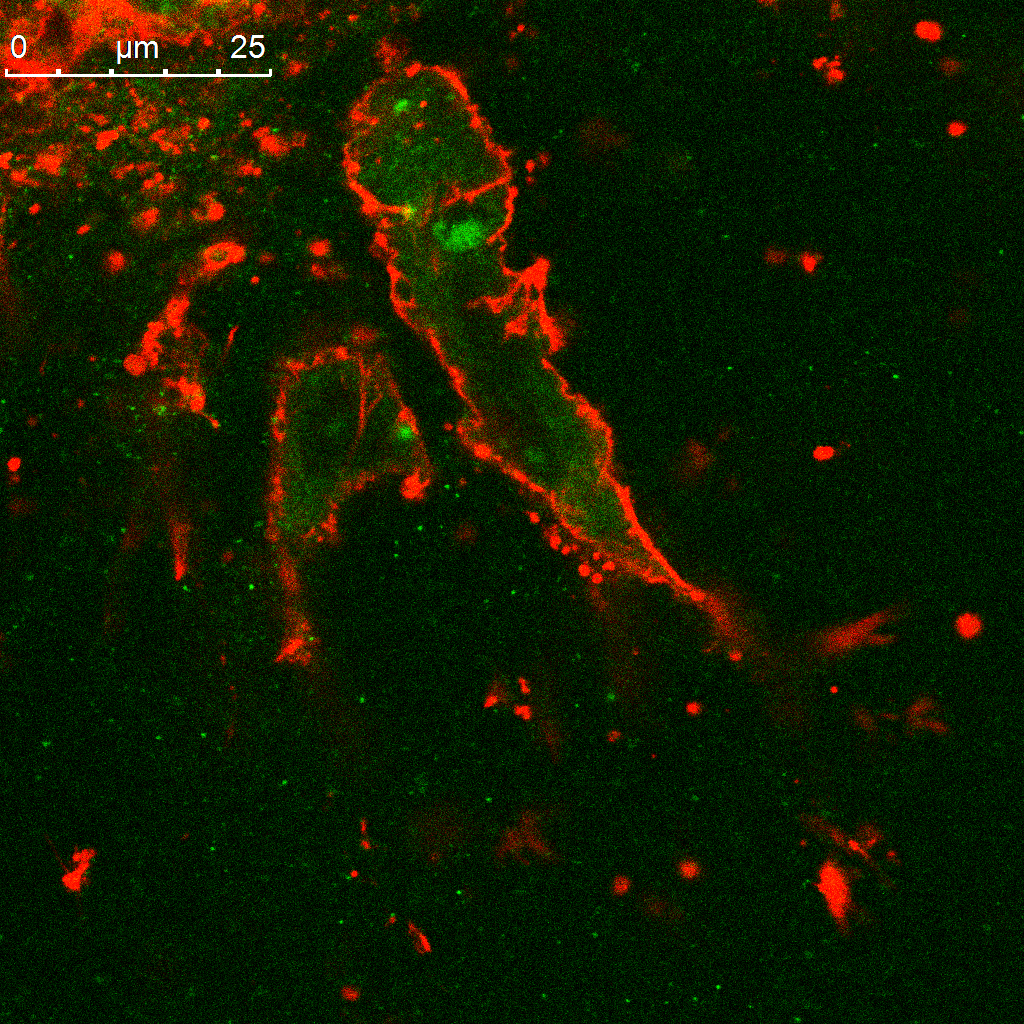

Supplement: S2 Dataset — Images in TIFF format. The measurements (performed using ImageJ) are given in file “cellareas.xlsx”. (ZIP) [file pone.0159478.s007.zip › sproutende ECs/Series035_z15.tif]

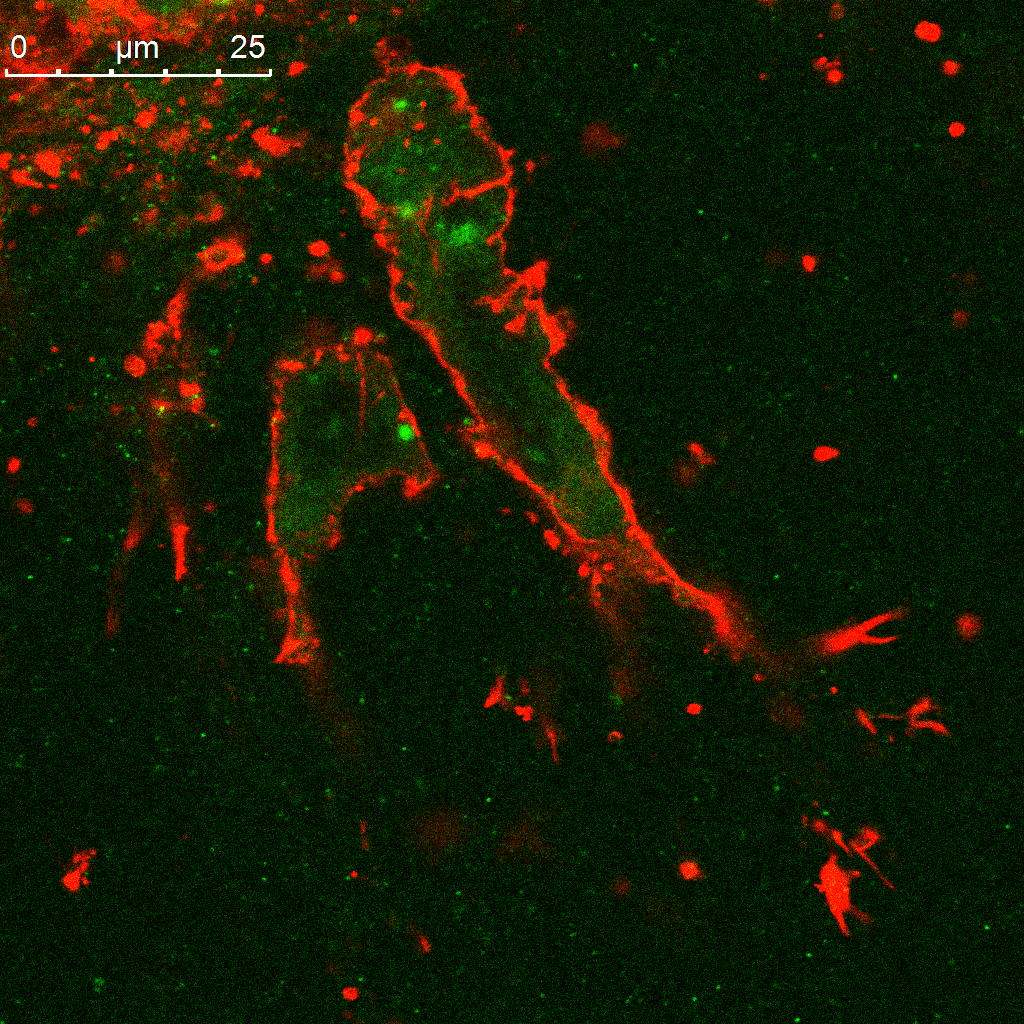

Supplement: S2 Dataset — Images in TIFF format. The measurements (performed using ImageJ) are given in file “cellareas.xlsx”. (ZIP) [file pone.0159478.s007.zip › sproutende ECs/Series035_z16.tif]

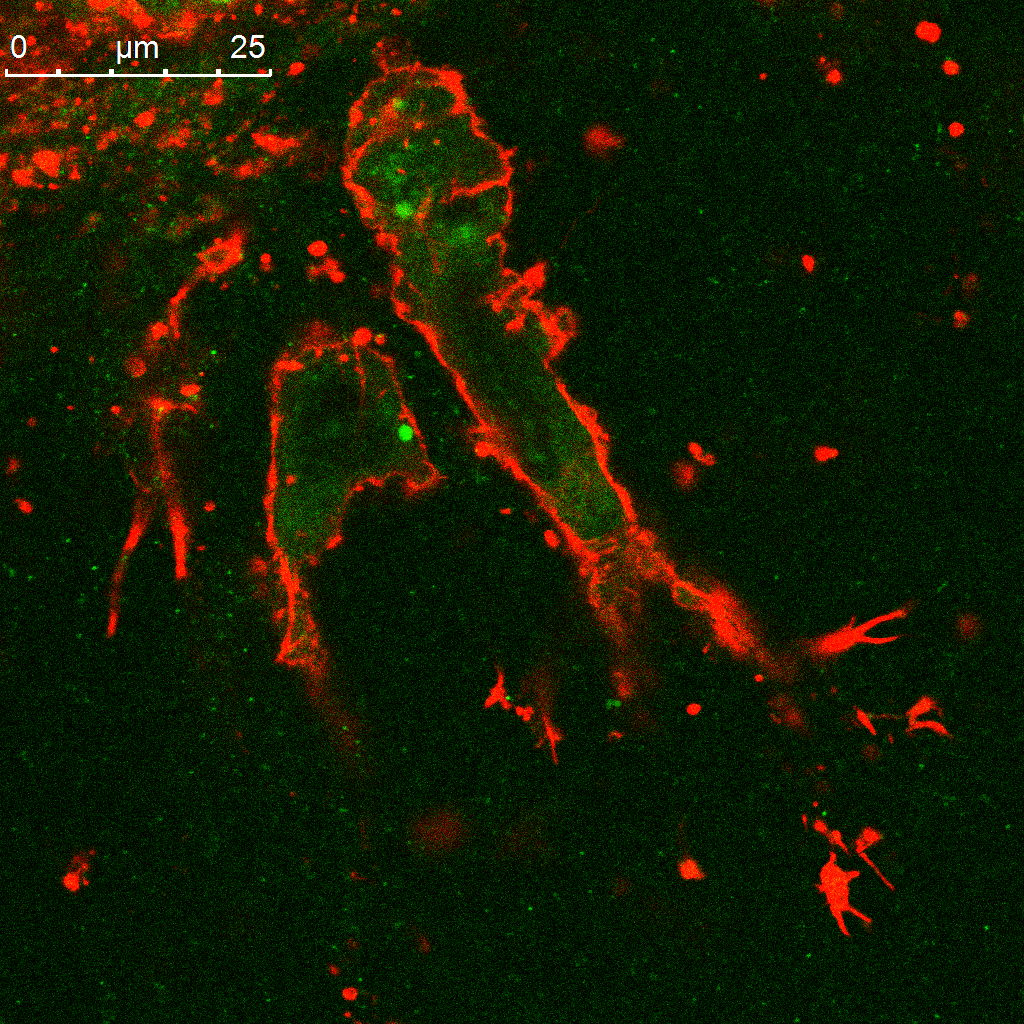

Supplement: S2 Dataset — Images in TIFF format. The measurements (performed using ImageJ) are given in file “cellareas.xlsx”. (ZIP) [file pone.0159478.s007.zip › sproutende ECs/Series035_z17.tif]

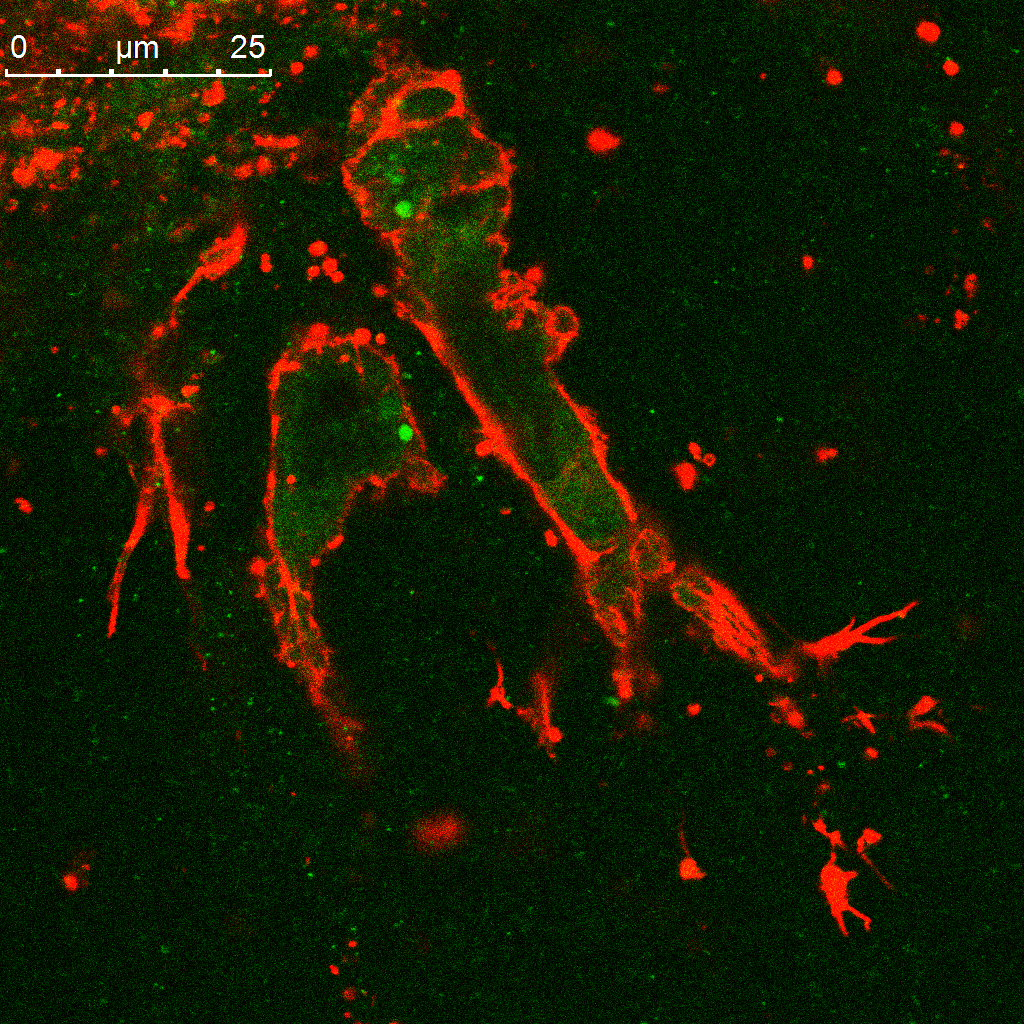

Supplement: S2 Dataset — Images in TIFF format. The measurements (performed using ImageJ) are given in file “cellareas.xlsx”. (ZIP) [file pone.0159478.s007.zip › sproutende ECs/Series035_z18.tif]

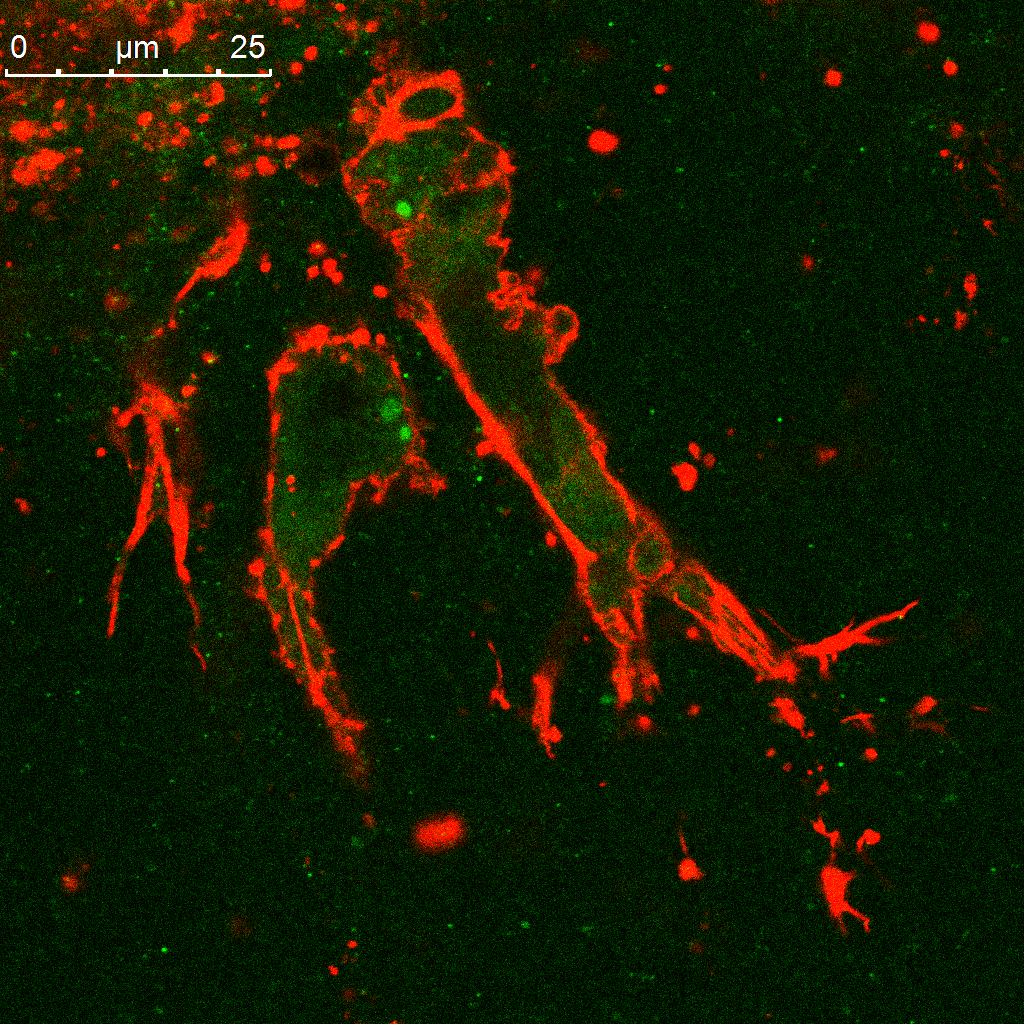

Supplement: S2 Dataset — Images in TIFF format. The measurements (performed using ImageJ) are given in file “cellareas.xlsx”. (ZIP) [file pone.0159478.s007.zip › sproutende ECs/Series035_z19.tif]

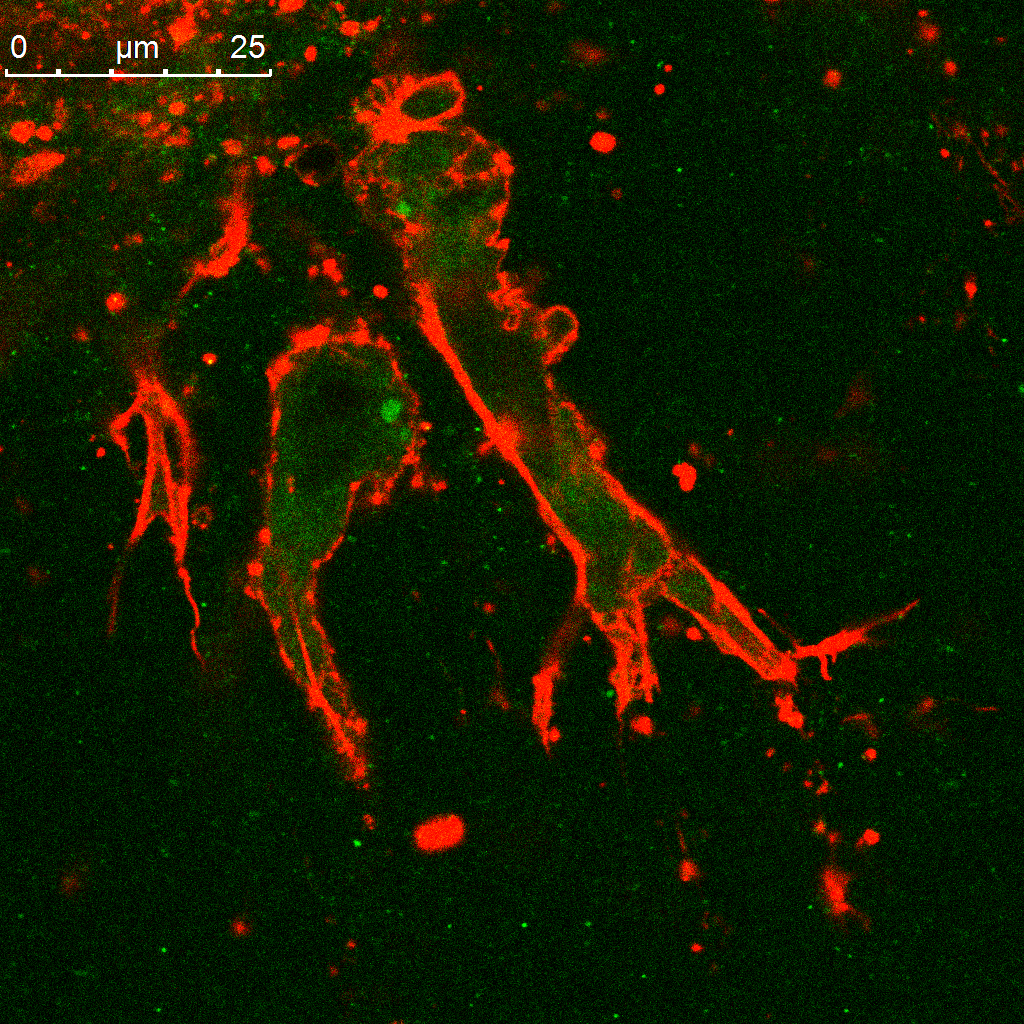

Supplement: S2 Dataset — Images in TIFF format. The measurements (performed using ImageJ) are given in file “cellareas.xlsx”. (ZIP) [file pone.0159478.s007.zip › sproutende ECs/Series035_z20.tif]

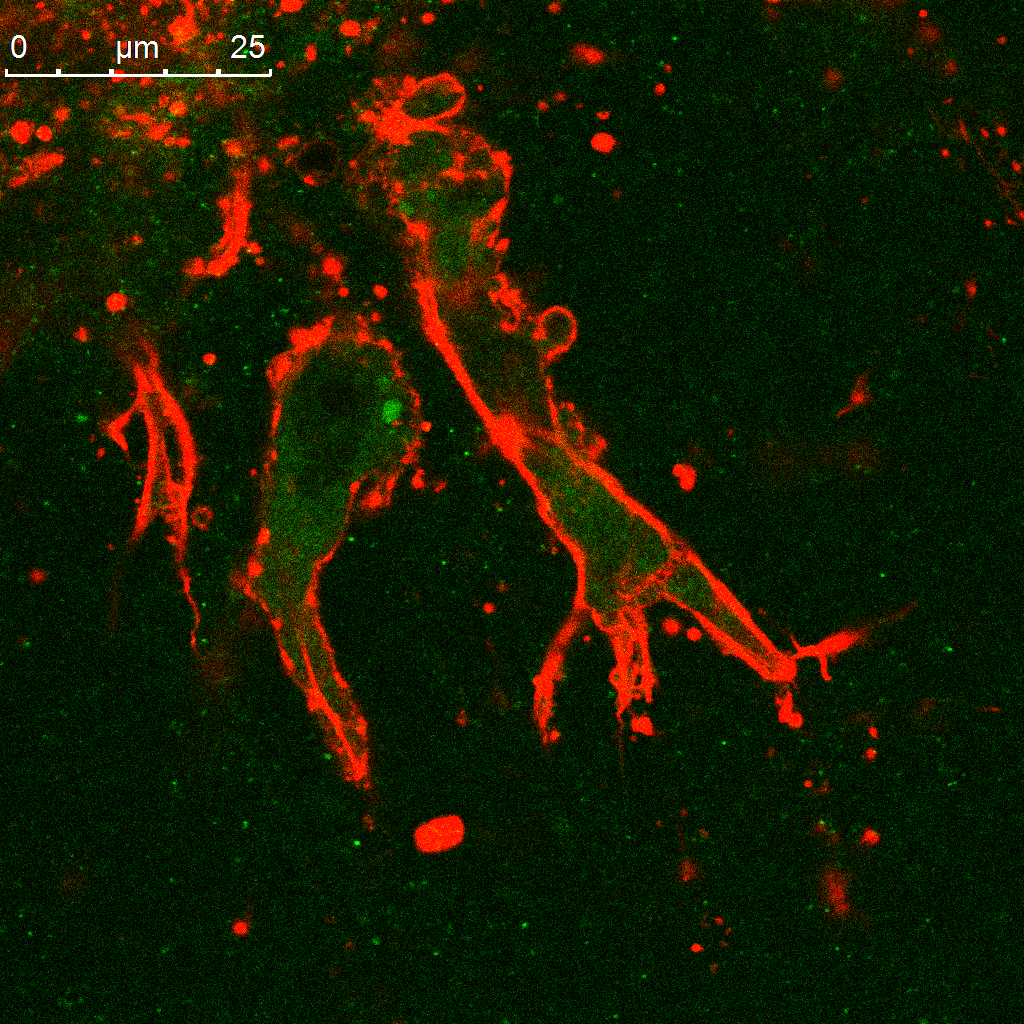

Supplement: S2 Dataset — Images in TIFF format. The measurements (performed using ImageJ) are given in file “cellareas.xlsx”. (ZIP) [file pone.0159478.s007.zip › sproutende ECs/Series035_z21.tif]

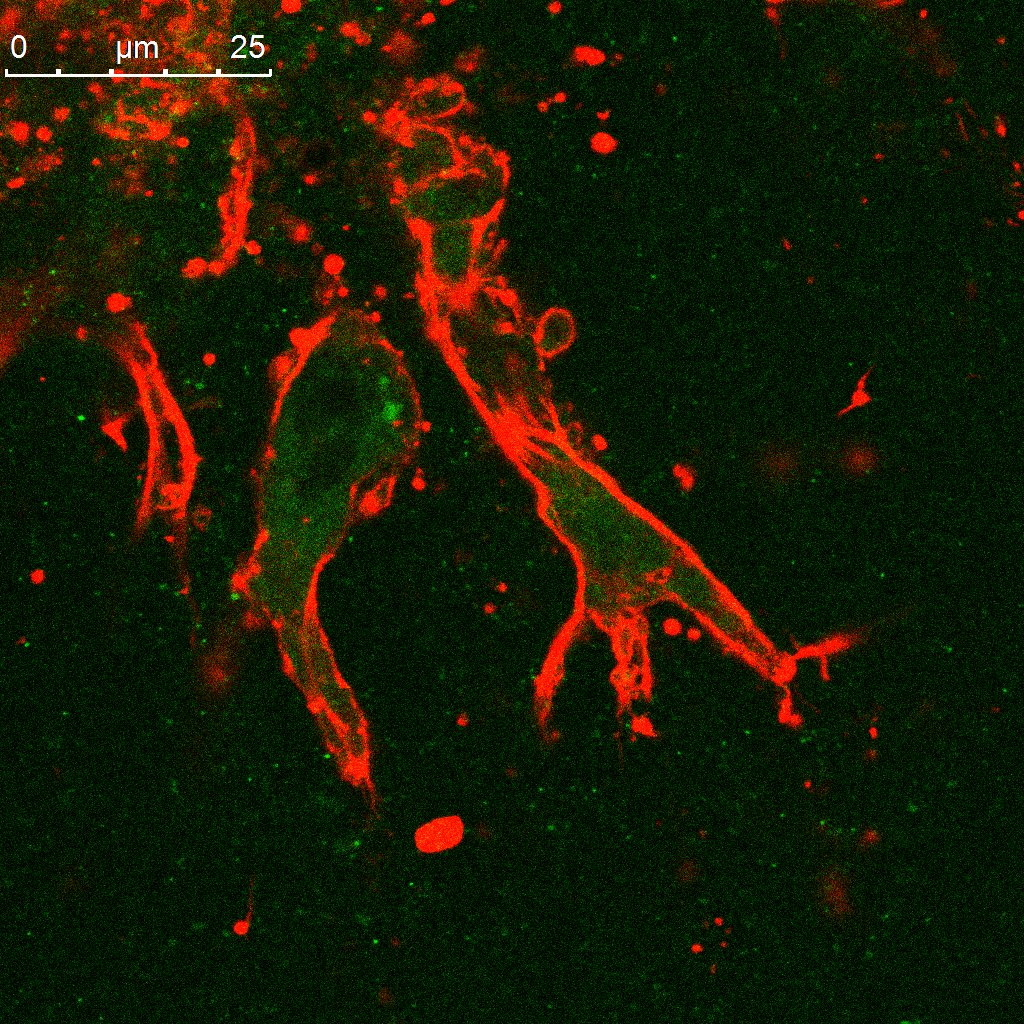

Supplement: S2 Dataset — Images in TIFF format. The measurements (performed using ImageJ) are given in file “cellareas.xlsx”. (ZIP) [file pone.0159478.s007.zip › sproutende ECs/Series035_z22.tif]

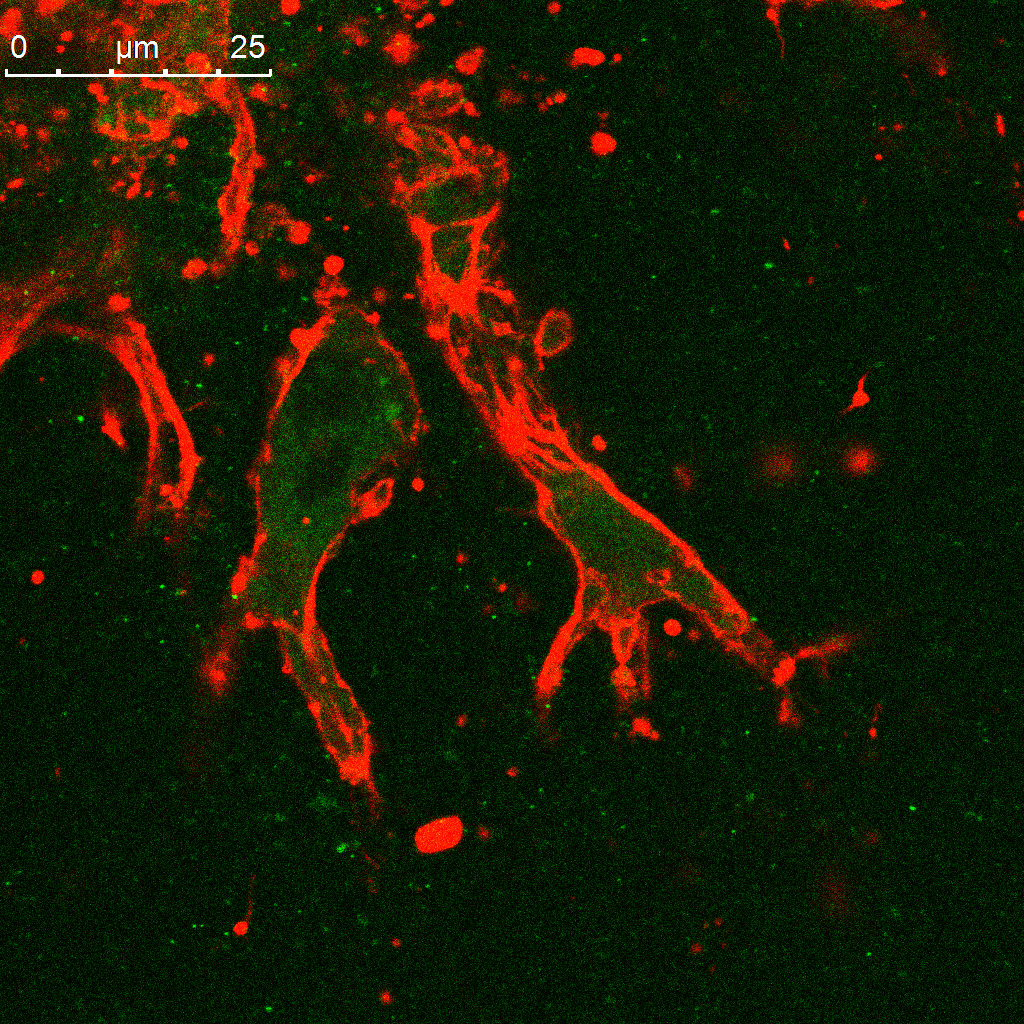

Supplement: S2 Dataset — Images in TIFF format. The measurements (performed using ImageJ) are given in file “cellareas.xlsx”. (ZIP) [file pone.0159478.s007.zip › sproutende ECs/Series035_z23.tif]

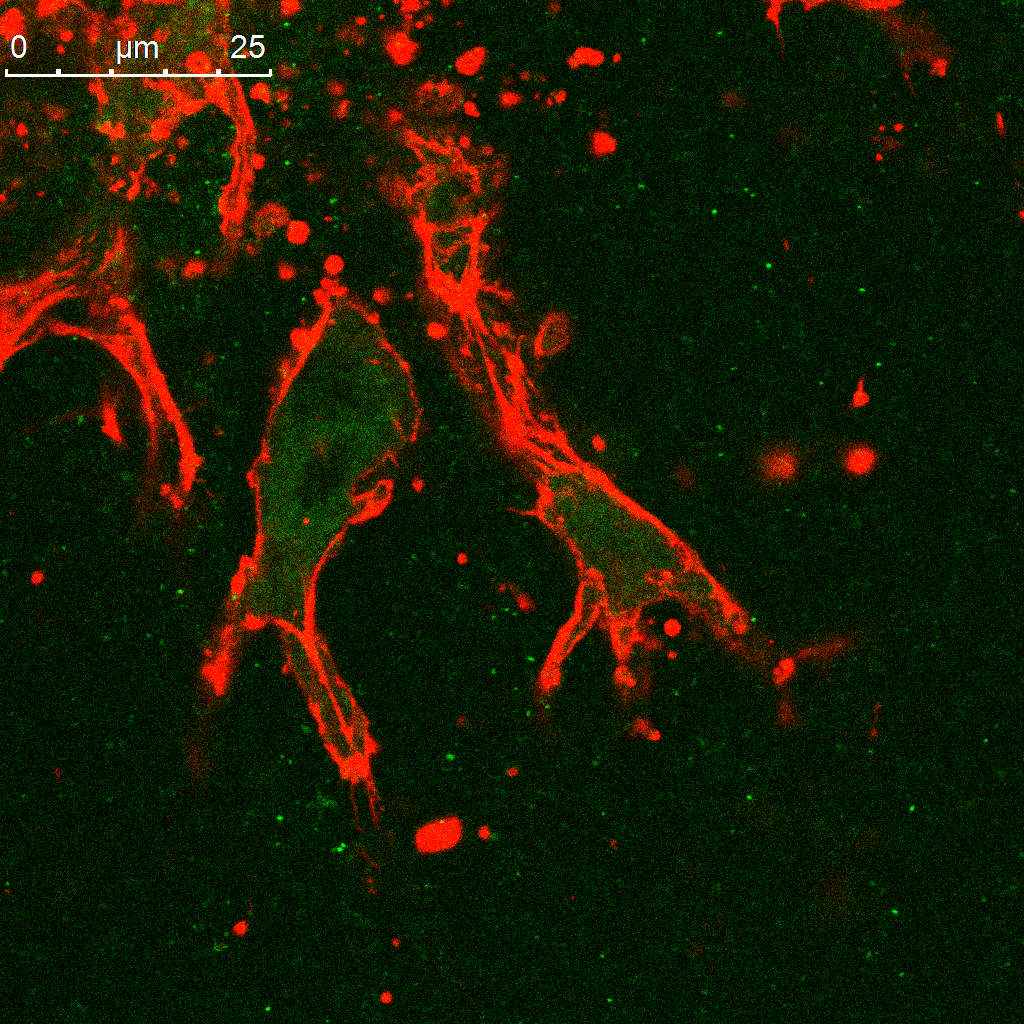

Supplement: S2 Dataset — Images in TIFF format. The measurements (performed using ImageJ) are given in file “cellareas.xlsx”. (ZIP) [file pone.0159478.s007.zip › sproutende ECs/Series035_z24.tif]

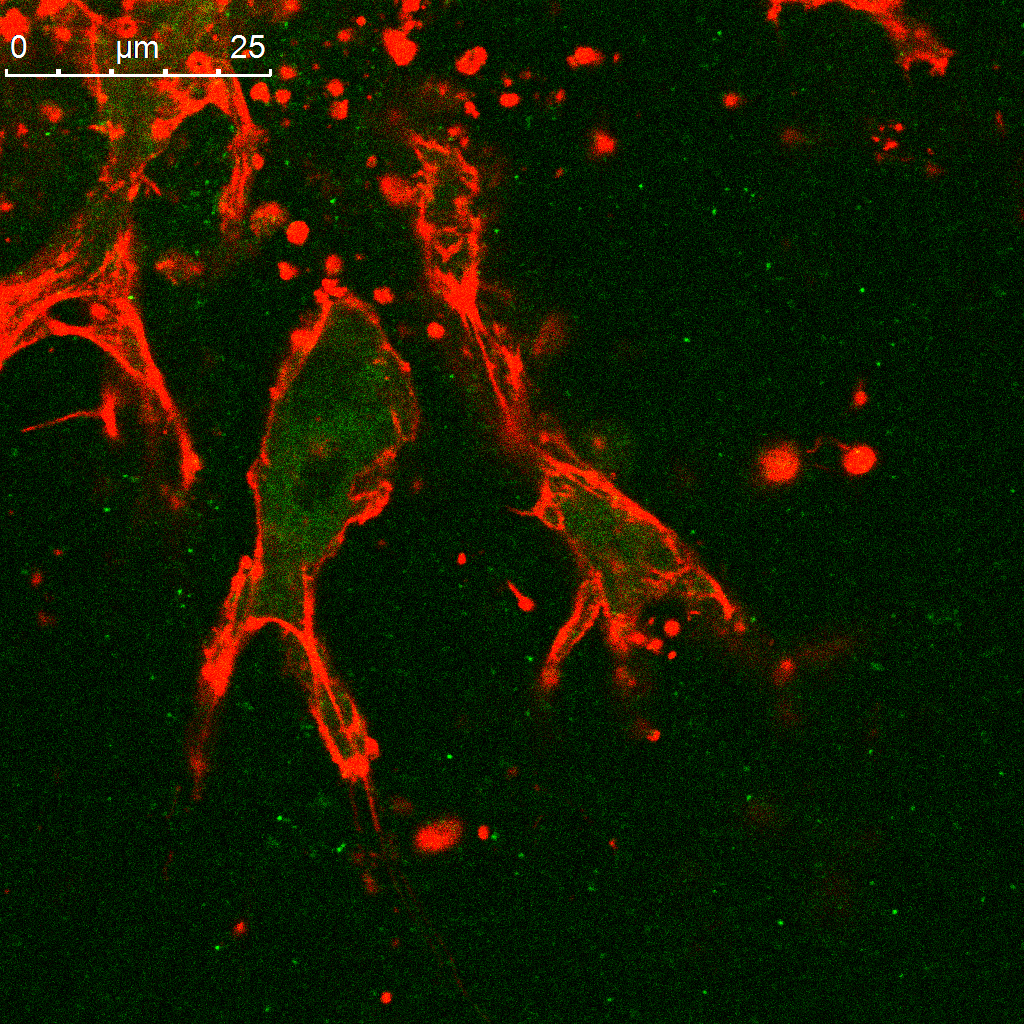

Supplement: S2 Dataset — Images in TIFF format. The measurements (performed using ImageJ) are given in file “cellareas.xlsx”. (ZIP) [file pone.0159478.s007.zip › sproutende ECs/Series035_z25.tif]

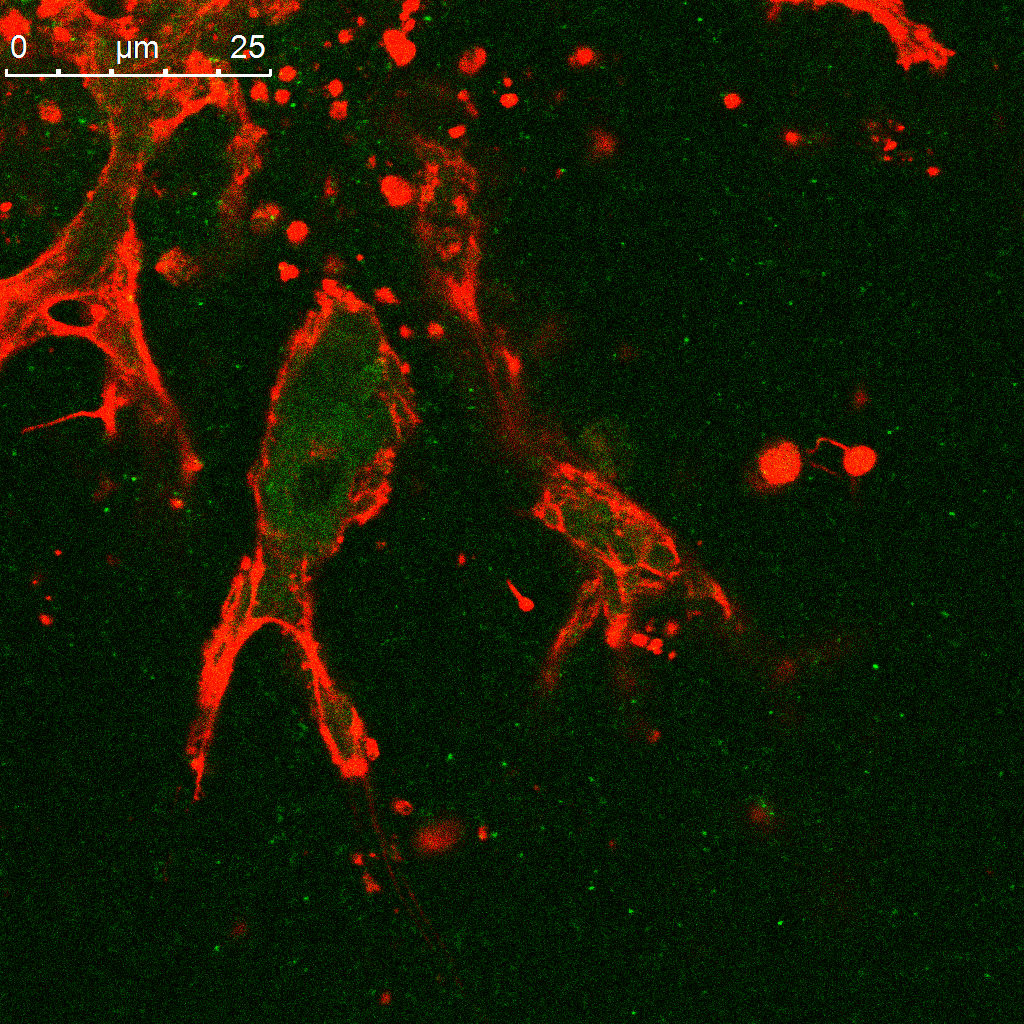

Supplement: S2 Dataset — Images in TIFF format. The measurements (performed using ImageJ) are given in file “cellareas.xlsx”. (ZIP) [file pone.0159478.s007.zip › sproutende ECs/Series035_z26.tif]

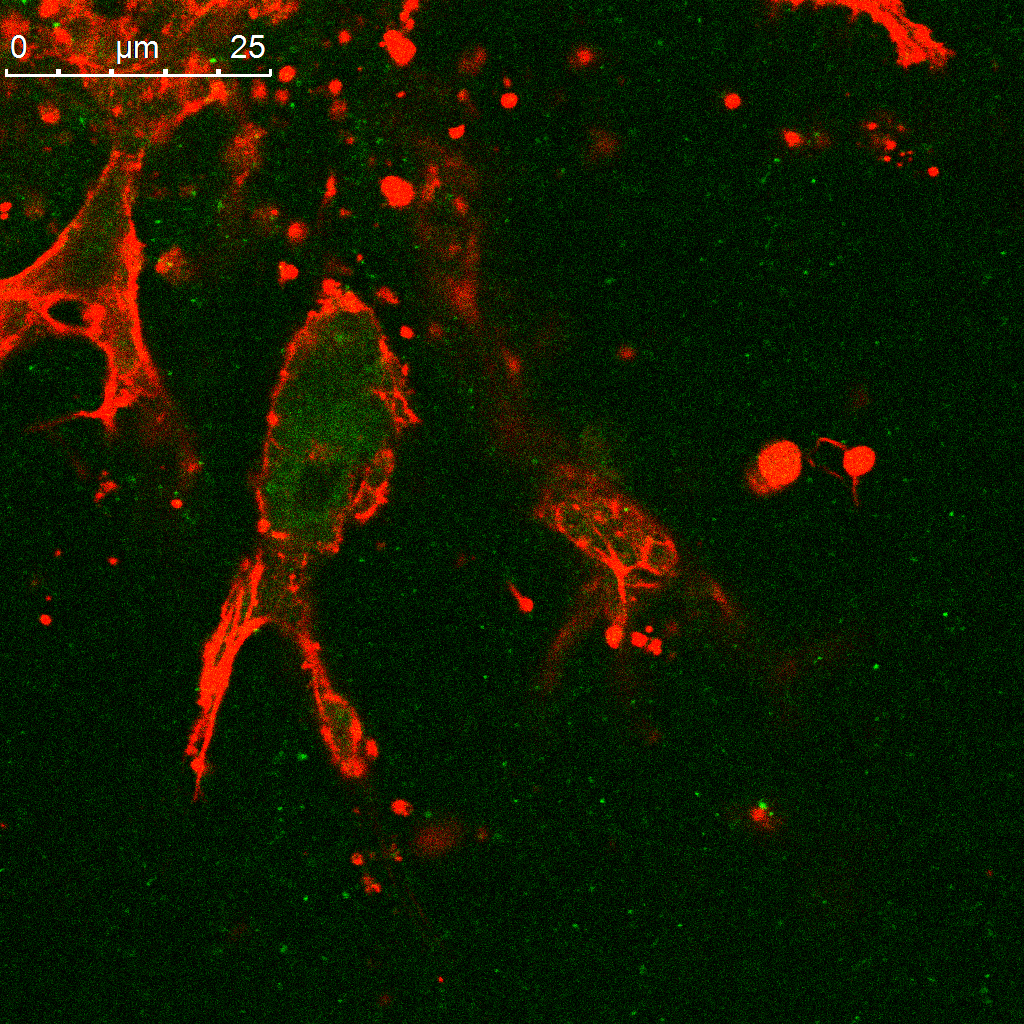

Supplement: S2 Dataset — Images in TIFF format. The measurements (performed using ImageJ) are given in file “cellareas.xlsx”. (ZIP) [file pone.0159478.s007.zip › sproutende ECs/Series035_z27.tif]

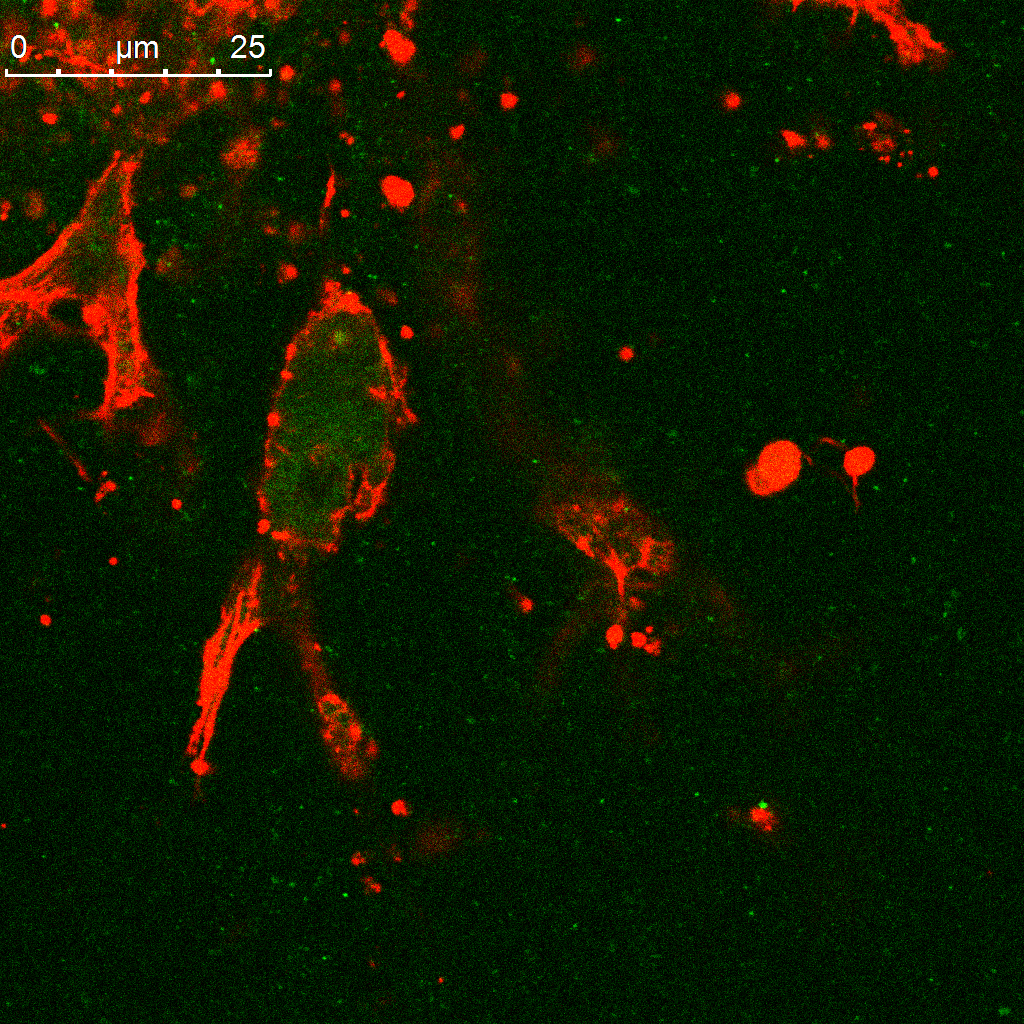

Supplement: S2 Dataset — Images in TIFF format. The measurements (performed using ImageJ) are given in file “cellareas.xlsx”. (ZIP) [file pone.0159478.s007.zip › sproutende ECs/Series035_z28.tif]

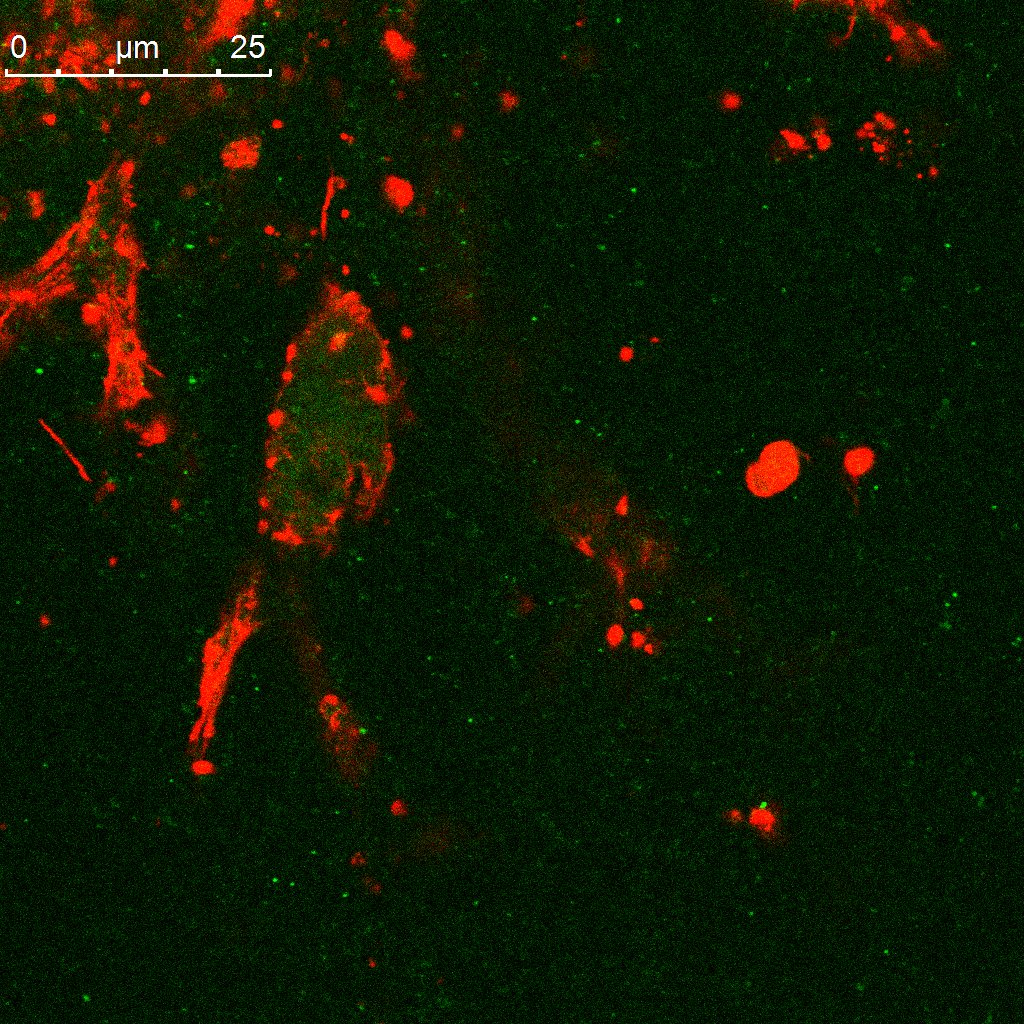

Supplement: S2 Dataset — Images in TIFF format. The measurements (performed using ImageJ) are given in file “cellareas.xlsx”. (ZIP) [file pone.0159478.s007.zip › sproutende ECs/Series035_z29.tif]

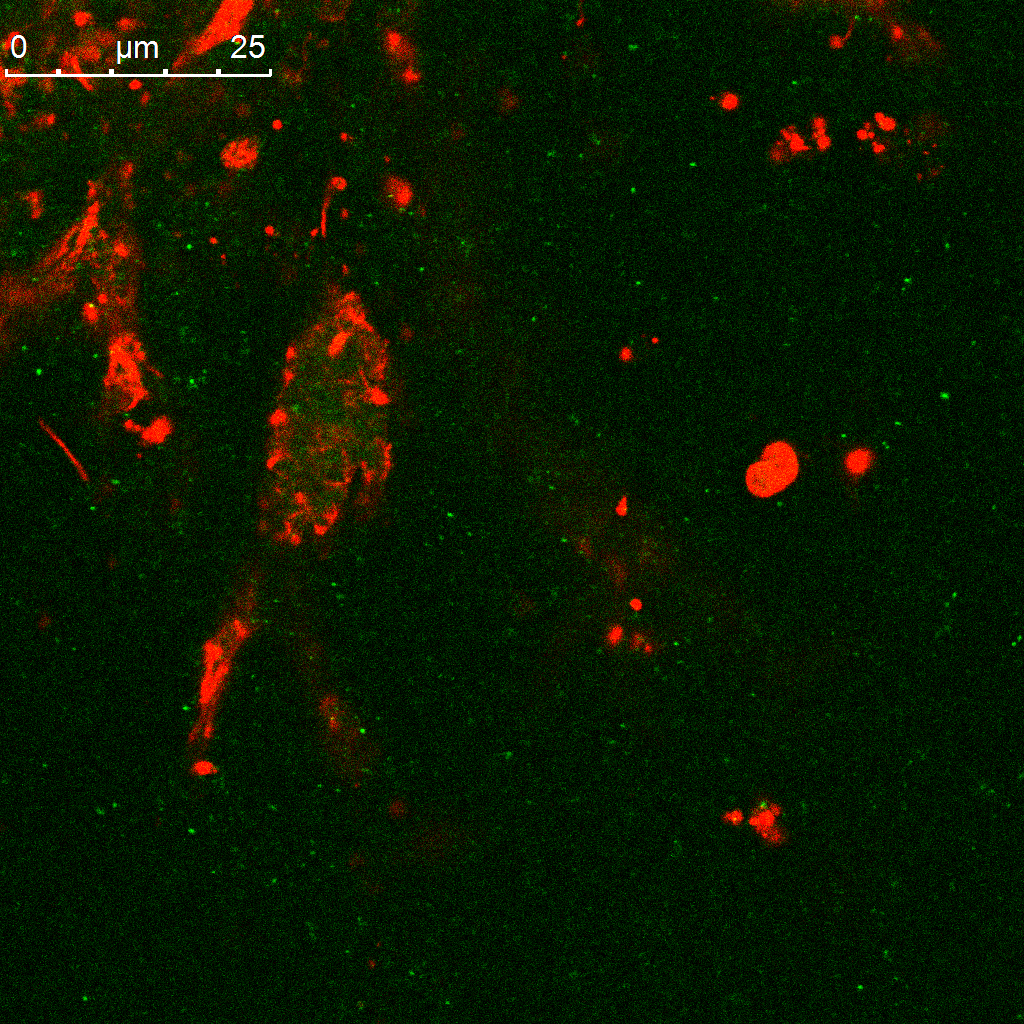

Supplement: S2 Dataset — Images in TIFF format. The measurements (performed using ImageJ) are given in file “cellareas.xlsx”. (ZIP) [file pone.0159478.s007.zip › sproutende ECs/Series035_z30.tif]

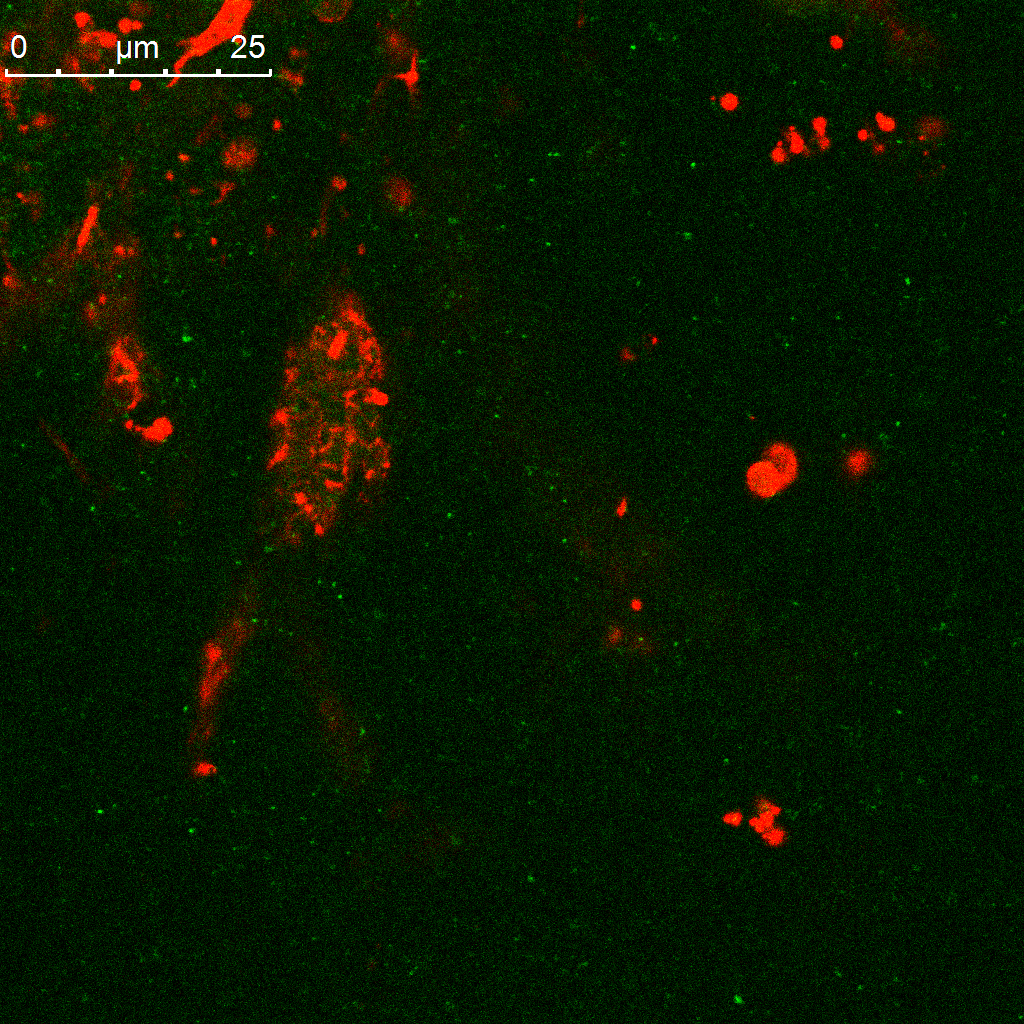

Supplement: S2 Dataset — Images in TIFF format. The measurements (performed using ImageJ) are given in file “cellareas.xlsx”. (ZIP) [file pone.0159478.s007.zip › sproutende ECs/Series035_z31.tif]

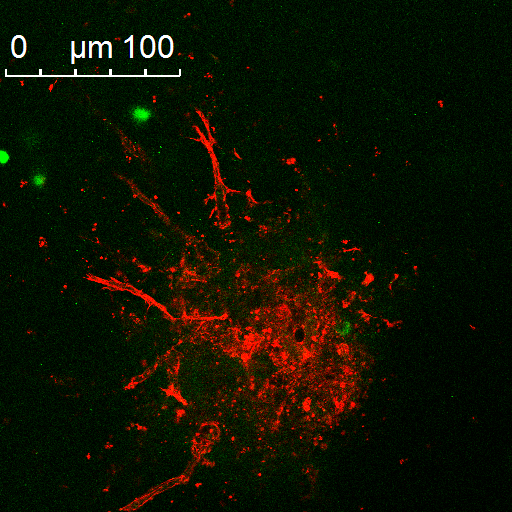

Supplement: S2 Dataset — Images in TIFF format. The measurements (performed using ImageJ) are given in file “cellareas.xlsx”. (ZIP) [file pone.0159478.s007.zip › sproutende ECs/Series037_z00.tif]

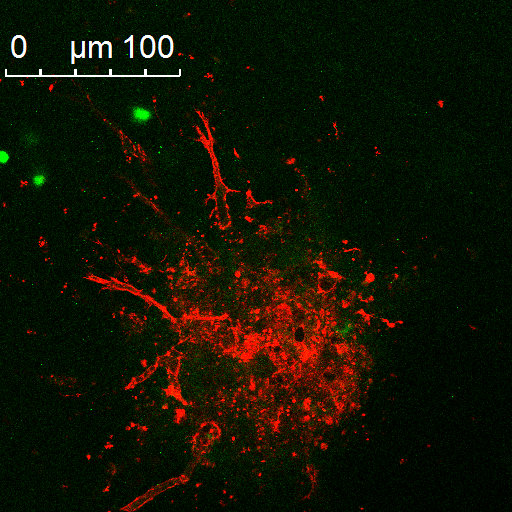

Supplement: S2 Dataset — Images in TIFF format. The measurements (performed using ImageJ) are given in file “cellareas.xlsx”. (ZIP) [file pone.0159478.s007.zip › sproutende ECs/Series037_z01.tif]

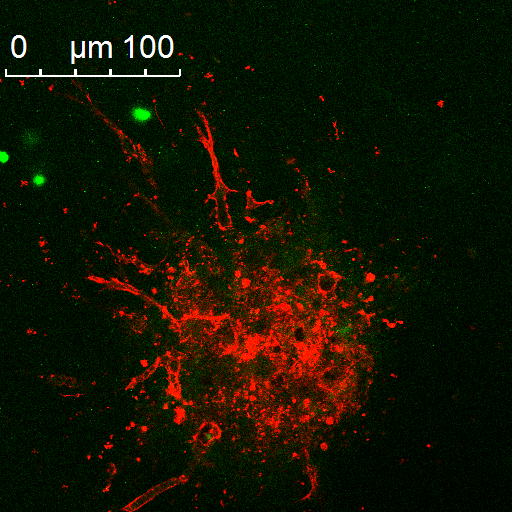

Supplement: S2 Dataset — Images in TIFF format. The measurements (performed using ImageJ) are given in file “cellareas.xlsx”. (ZIP) [file pone.0159478.s007.zip › sproutende ECs/Series037_z02.tif]

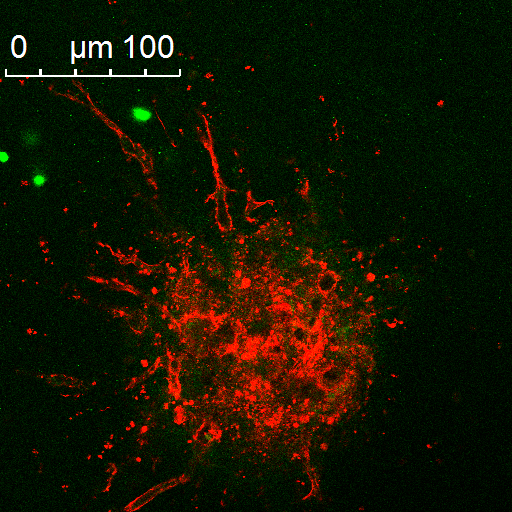

Supplement: S2 Dataset — Images in TIFF format. The measurements (performed using ImageJ) are given in file “cellareas.xlsx”. (ZIP) [file pone.0159478.s007.zip › sproutende ECs/Series037_z03.tif]

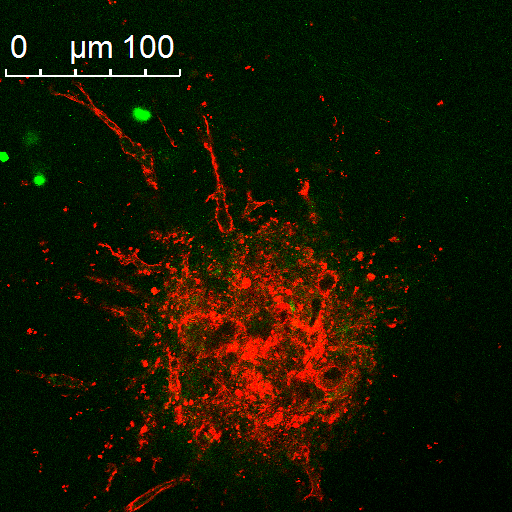

Supplement: S2 Dataset — Images in TIFF format. The measurements (performed using ImageJ) are given in file “cellareas.xlsx”. (ZIP) [file pone.0159478.s007.zip › sproutende ECs/Series037_z04.tif]

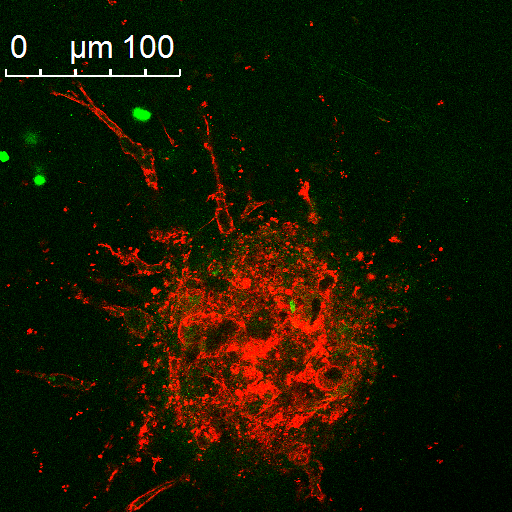

Supplement: S2 Dataset — Images in TIFF format. The measurements (performed using ImageJ) are given in file “cellareas.xlsx”. (ZIP) [file pone.0159478.s007.zip › sproutende ECs/Series037_z05.tif]

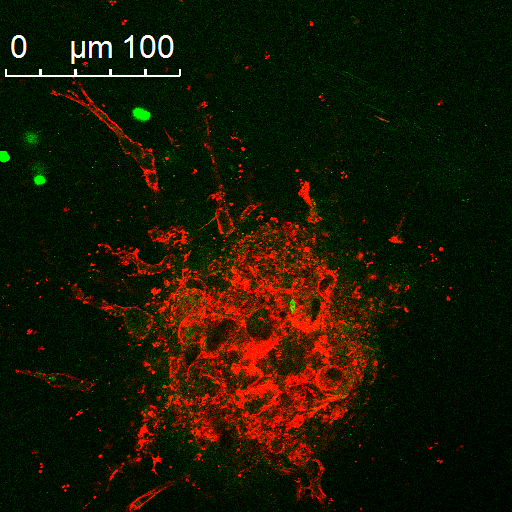

Supplement: S2 Dataset — Images in TIFF format. The measurements (performed using ImageJ) are given in file “cellareas.xlsx”. (ZIP) [file pone.0159478.s007.zip › sproutende ECs/Series037_z06.tif]

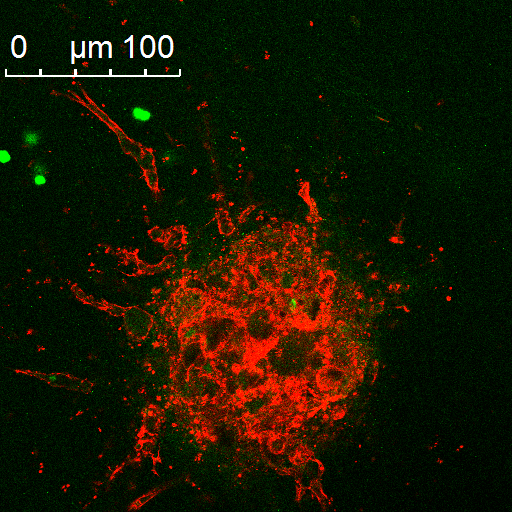

Supplement: S2 Dataset — Images in TIFF format. The measurements (performed using ImageJ) are given in file “cellareas.xlsx”. (ZIP) [file pone.0159478.s007.zip › sproutende ECs/Series037_z07.tif]

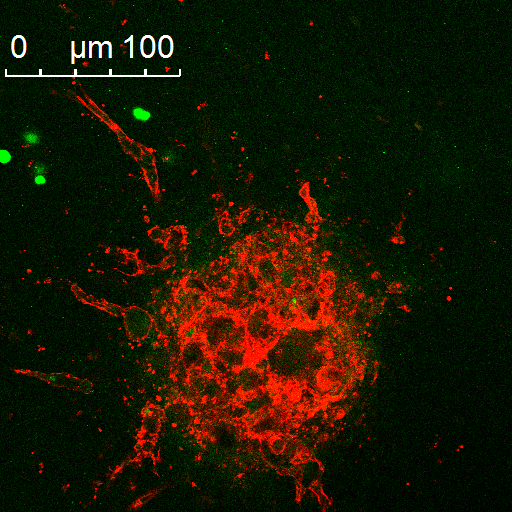

Supplement: S2 Dataset — Images in TIFF format. The measurements (performed using ImageJ) are given in file “cellareas.xlsx”. (ZIP) [file pone.0159478.s007.zip › sproutende ECs/Series037_z08.tif]

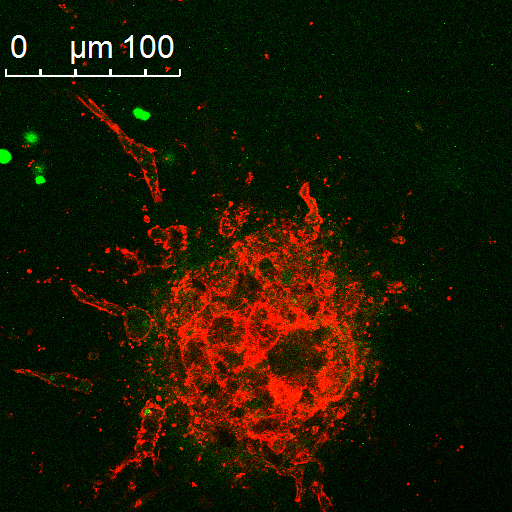

Supplement: S2 Dataset — Images in TIFF format. The measurements (performed using ImageJ) are given in file “cellareas.xlsx”. (ZIP) [file pone.0159478.s007.zip › sproutende ECs/Series037_z09.tif]

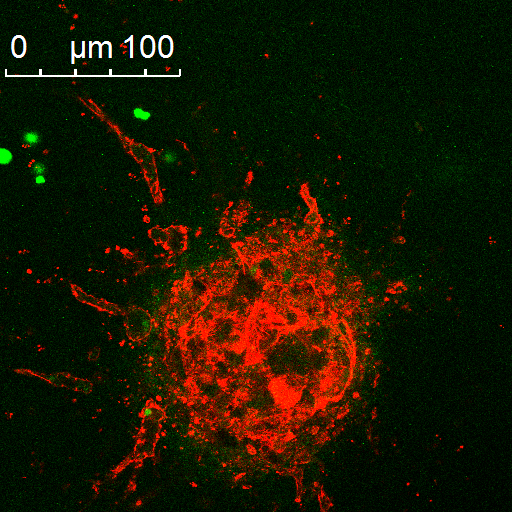

Supplement: S2 Dataset — Images in TIFF format. The measurements (performed using ImageJ) are given in file “cellareas.xlsx”. (ZIP) [file pone.0159478.s007.zip › sproutende ECs/Series037_z10.tif]

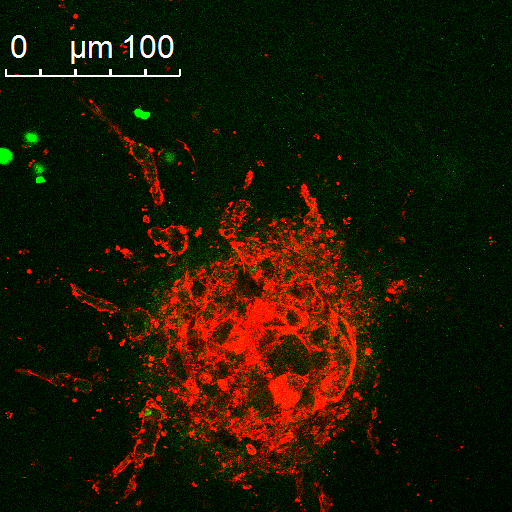

Supplement: S2 Dataset — Images in TIFF format. The measurements (performed using ImageJ) are given in file “cellareas.xlsx”. (ZIP) [file pone.0159478.s007.zip › sproutende ECs/Series037_z11.tif]

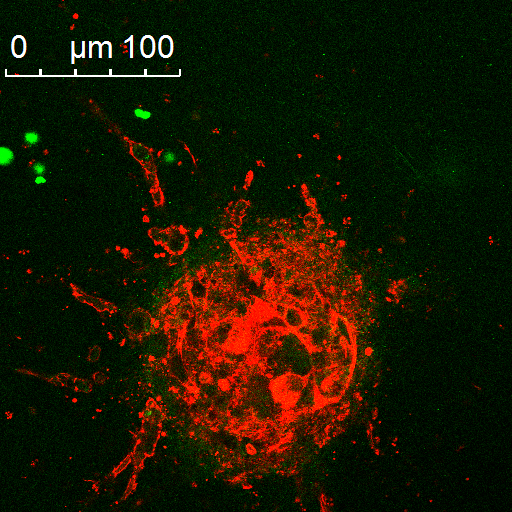

Supplement: S2 Dataset — Images in TIFF format. The measurements (performed using ImageJ) are given in file “cellareas.xlsx”. (ZIP) [file pone.0159478.s007.zip › sproutende ECs/Series037_z12.tif]

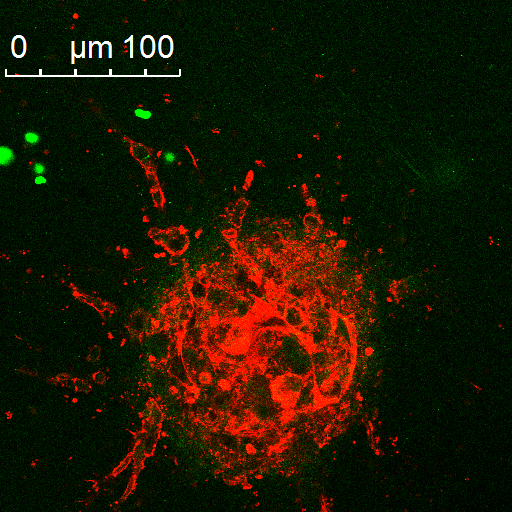

Supplement: S2 Dataset — Images in TIFF format. The measurements (performed using ImageJ) are given in file “cellareas.xlsx”. (ZIP) [file pone.0159478.s007.zip › sproutende ECs/Series037_z13.tif]

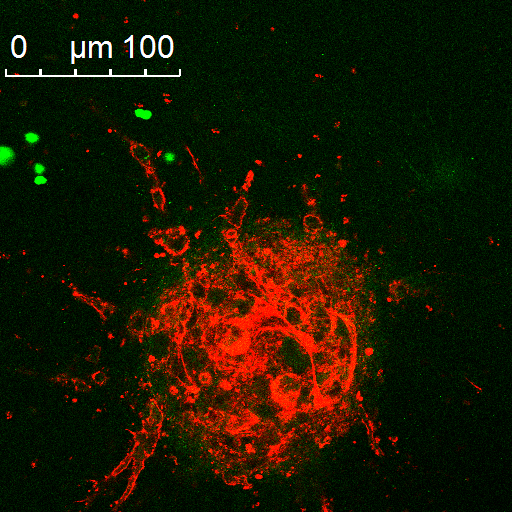

Supplement: S2 Dataset — Images in TIFF format. The measurements (performed using ImageJ) are given in file “cellareas.xlsx”. (ZIP) [file pone.0159478.s007.zip › sproutende ECs/Series037_z14.tif]

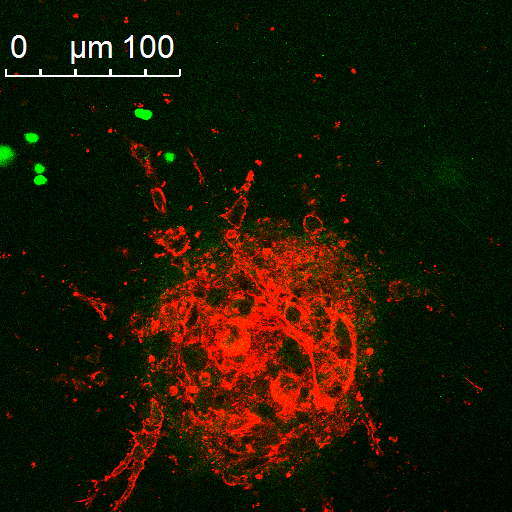

Supplement: S2 Dataset — Images in TIFF format. The measurements (performed using ImageJ) are given in file “cellareas.xlsx”. (ZIP) [file pone.0159478.s007.zip › sproutende ECs/Series037_z15.tif]

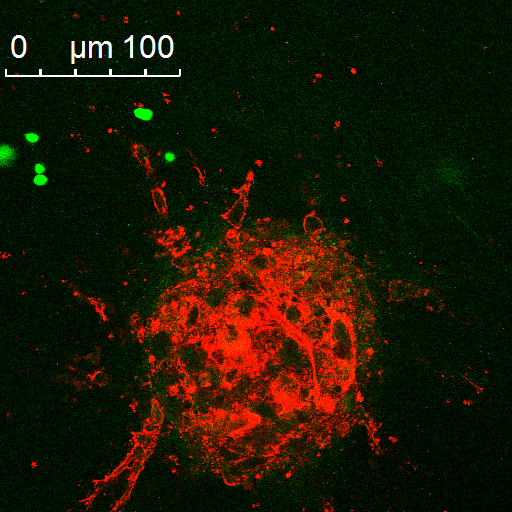

Supplement: S2 Dataset — Images in TIFF format. The measurements (performed using ImageJ) are given in file “cellareas.xlsx”. (ZIP) [file pone.0159478.s007.zip › sproutende ECs/Series037_z16.tif]

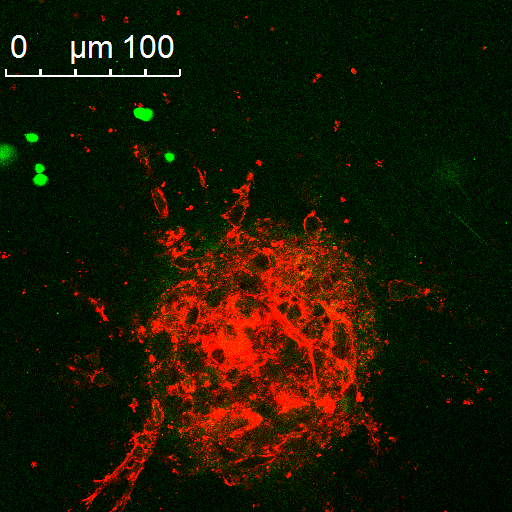

Supplement: S2 Dataset — Images in TIFF format. The measurements (performed using ImageJ) are given in file “cellareas.xlsx”. (ZIP) [file pone.0159478.s007.zip › sproutende ECs/Series037_z17.tif]

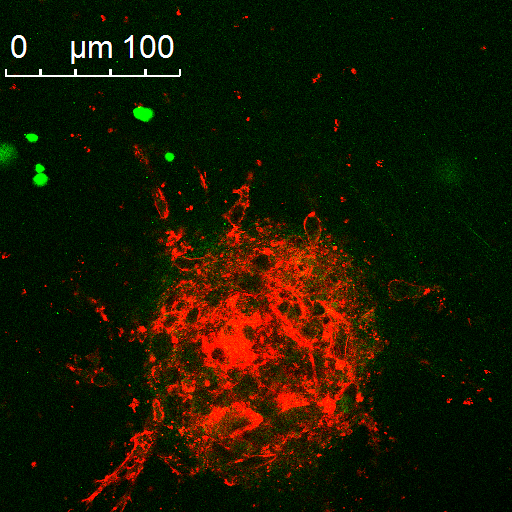

Supplement: S2 Dataset — Images in TIFF format. The measurements (performed using ImageJ) are given in file “cellareas.xlsx”. (ZIP) [file pone.0159478.s007.zip › sproutende ECs/Series037_z18.tif]

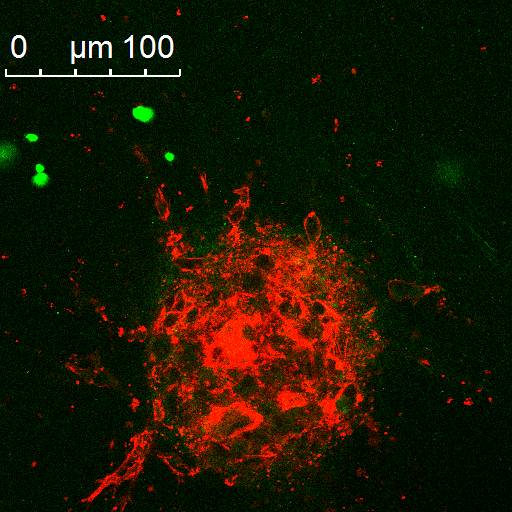

Supplement: S2 Dataset — Images in TIFF format. The measurements (performed using ImageJ) are given in file “cellareas.xlsx”. (ZIP) [file pone.0159478.s007.zip › sproutende ECs/Series037_z19.tif]

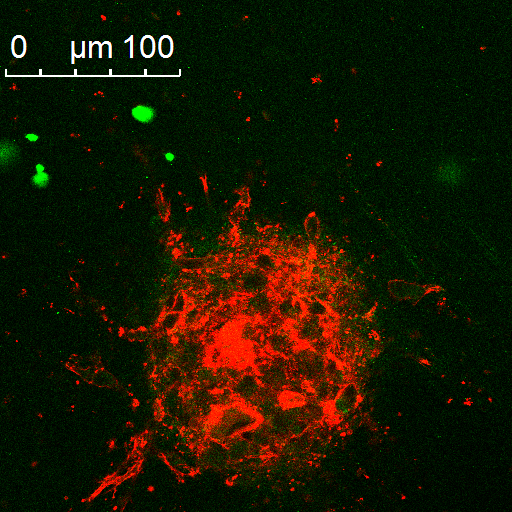

Supplement: S2 Dataset — Images in TIFF format. The measurements (performed using ImageJ) are given in file “cellareas.xlsx”. (ZIP) [file pone.0159478.s007.zip › sproutende ECs/Series037_z20.tif]

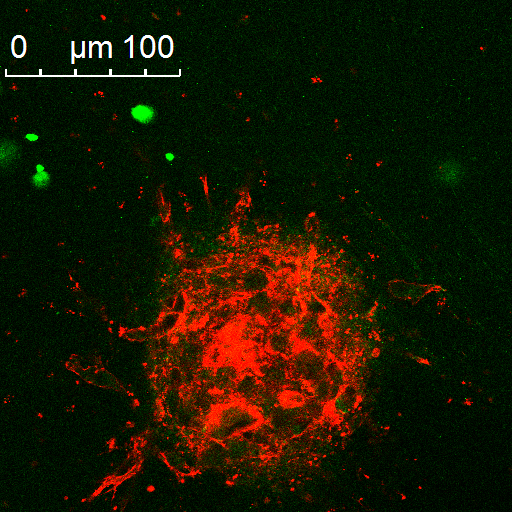

Supplement: S2 Dataset — Images in TIFF format. The measurements (performed using ImageJ) are given in file “cellareas.xlsx”. (ZIP) [file pone.0159478.s007.zip › sproutende ECs/Series037_z21.tif]

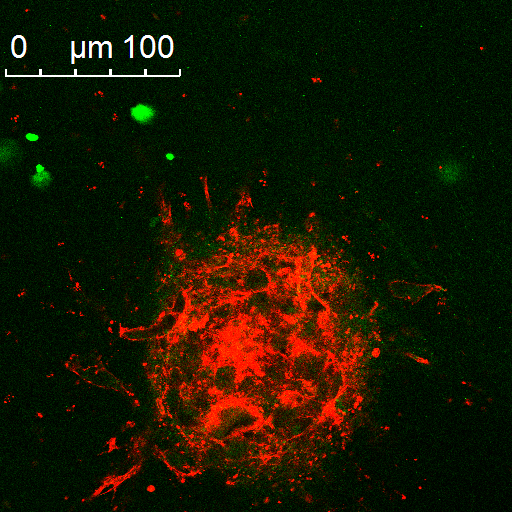

Supplement: S2 Dataset — Images in TIFF format. The measurements (performed using ImageJ) are given in file “cellareas.xlsx”. (ZIP) [file pone.0159478.s007.zip › sproutende ECs/Series037_z22.tif]

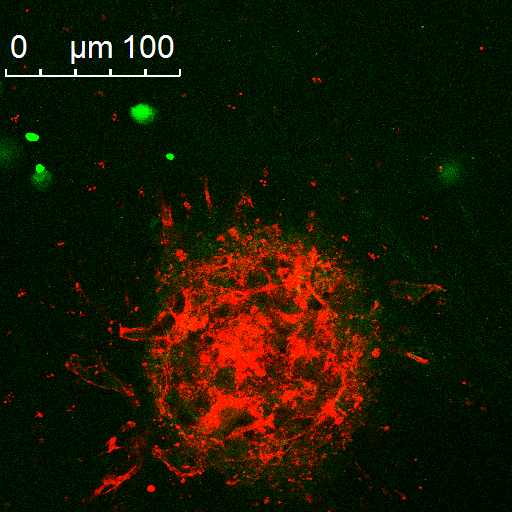

Supplement: S2 Dataset — Images in TIFF format. The measurements (performed using ImageJ) are given in file “cellareas.xlsx”. (ZIP) [file pone.0159478.s007.zip › sproutende ECs/Series037_z23.tif]

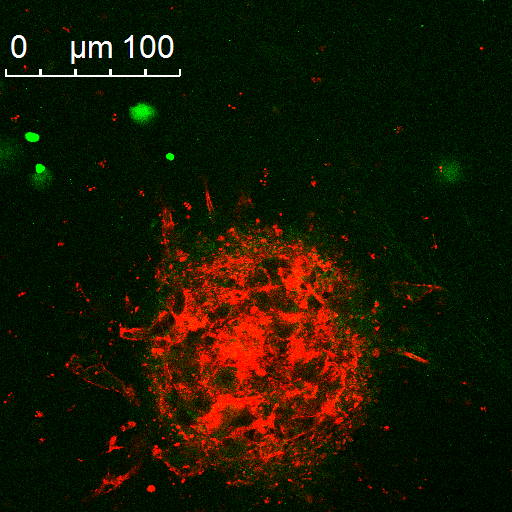

Supplement: S2 Dataset — Images in TIFF format. The measurements (performed using ImageJ) are given in file “cellareas.xlsx”. (ZIP) [file pone.0159478.s007.zip › sproutende ECs/Series037_z24.tif]

$J(\text{stalk}, \text{ECM}) = 5$

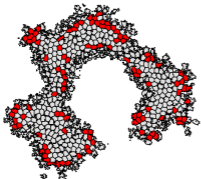

**A**

$J(\text{stalk}, \text{ECM}) = 10$

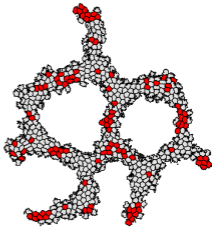

**B**

Supplement: S1 Fig — A stalk cells that adhere more strongly to the ECM than tip cells will engulf tip cells. B stalk cells that adhere slightly more to the ECM than tip cells do engulf tip cells, because chemotaxis has the same effect on tip and stalk cells. A-B are the results of a simulation of 10 000 MCS with 20% tip cells. (PDF) [file pone.0159478.s009.pdf]

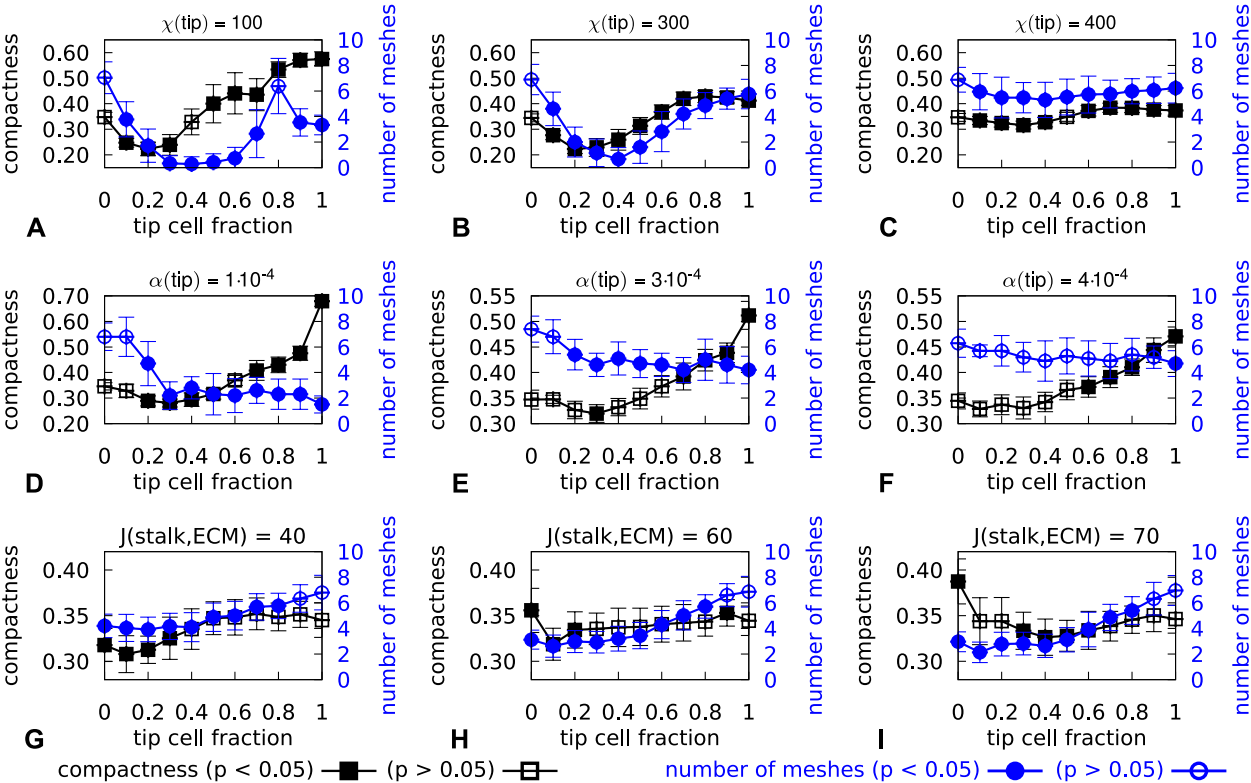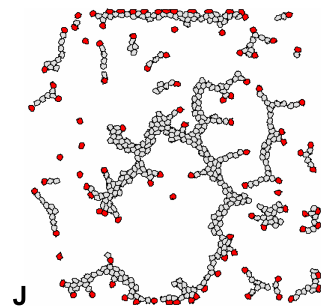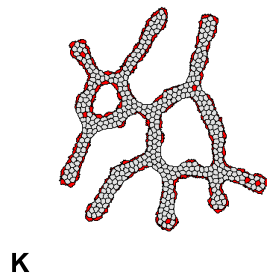

Supplement: S2 Fig — (A-C), tip cell chemoattractant secretion rate (D-F) and stalk-ECM adhesion (G-I). The morphometrics were obtained after 10 000 MCS and are the average of 50 simulations (error bars represent standard deviation). p-values were obtained with a Welch’s t-test for the null hypothesis that the mean of the sample is identical to that of a reference where all cells have the default properties. J the network disintegrates with χ(tip) = 100) and 20% tip cells. K tip cells over the network for J(stalk, ECM) = 70 and 20% tip cells. (PDF) [file pone.0159478.s010.pdf]

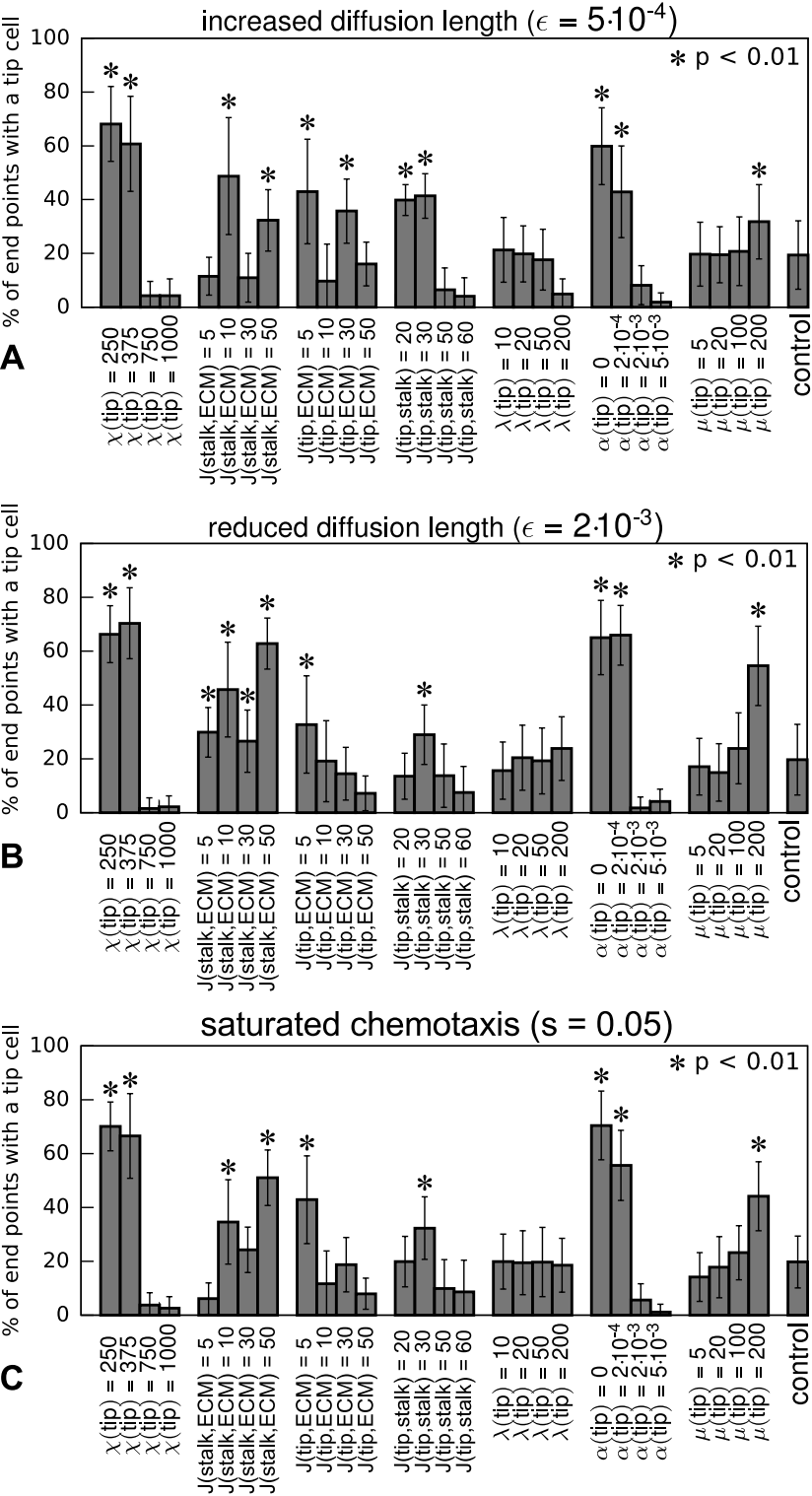

Supplement: S3 Fig — For A the decay rate was reduced, for B the decay rate was increased and for C receptor saturation was included in the model. The percentage of sprout tips occupied by at least one tip cell was calculated at 10 000 MCS. Error bars show the standard deviation over 50 simulations. In each simulation 20% of the cells were predefined as tip cells. For each simulation one tip cell parameter was changed, except for the control experiment where the nominal parameters were used for both tip and stalk cells. p-values were obtained with a one sided Welch’s t-test for the null hypothesis that the number of tip cells at the sprout tips is not larger than in the control simulation. (PDF) [file pone.0159478.s011.pdf]

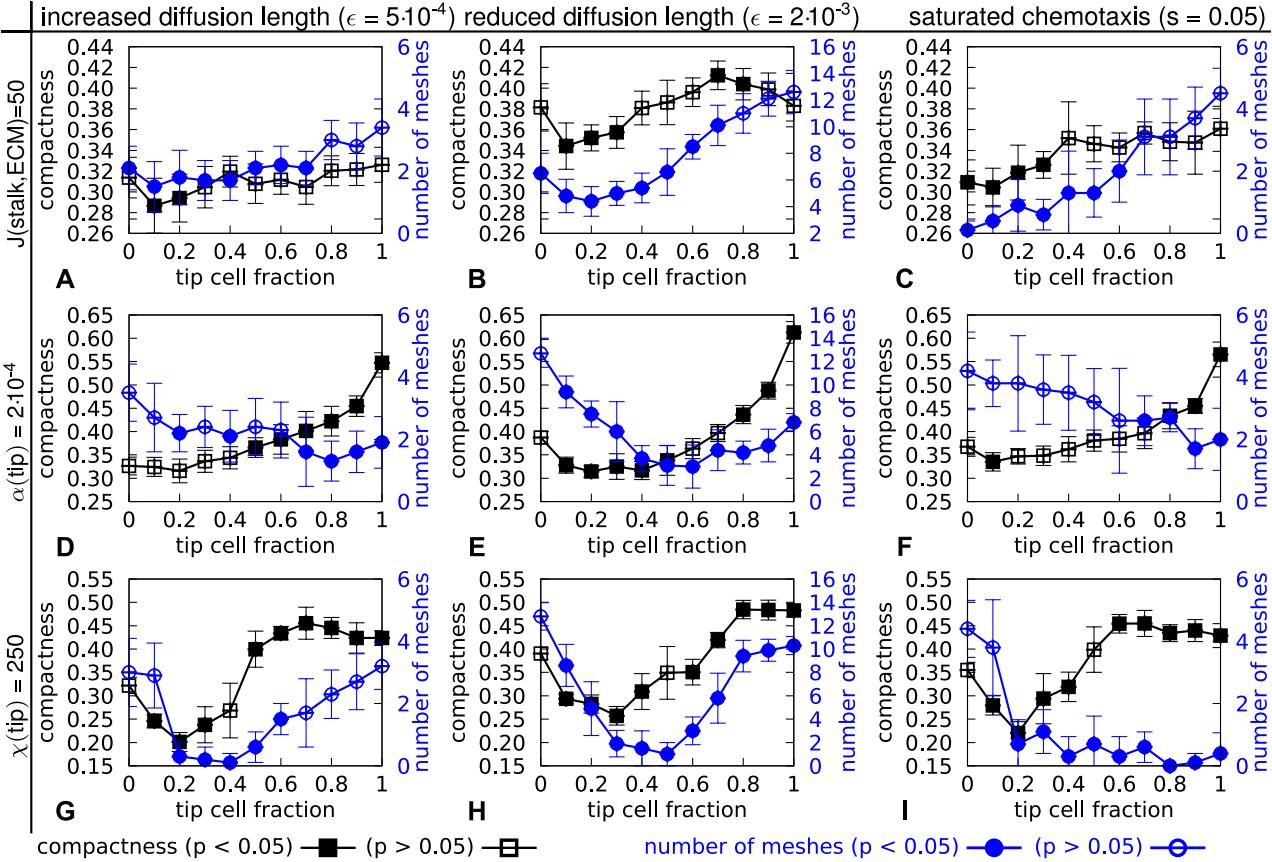

Supplement: S4 Fig — The morphometrics were obtained after 10 000 MCS and are the average of 10 simulations (error bars represent standard deviation). p-values were obtained with a Welch’s t-test for the null hypothesis that the mean of the sample is identical to that of a reference sample in which all cells have the default properties. (PDF) [file pone.0159478.s012.pdf]

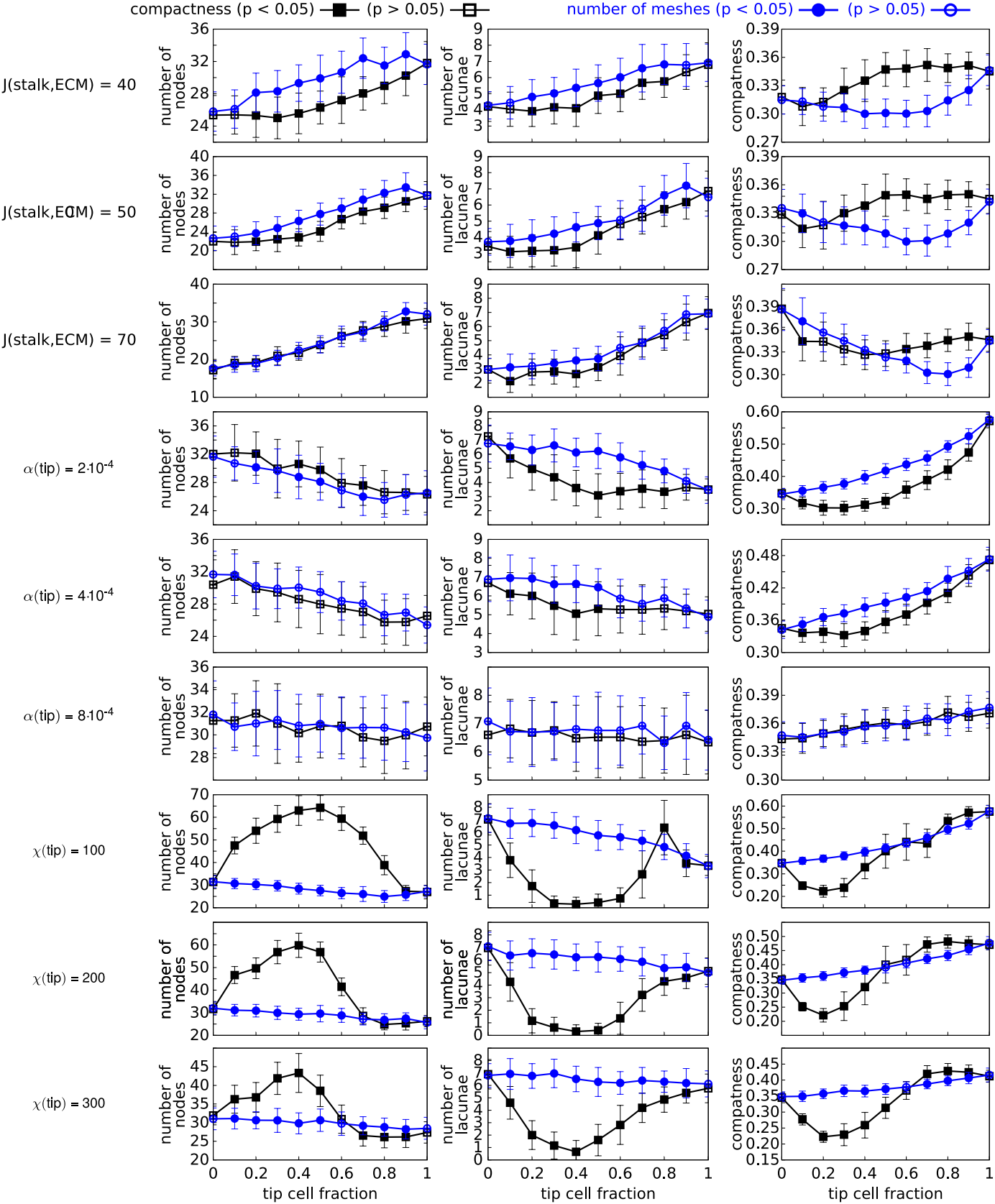

Supplement: S5 Fig — The morphometrics were calculated for 50 simulations at 10 000 MCS (error bars represent the standard deviation). p-values were obtained with a Welch’s t-test for the null hypothesis that the mean of mixed model and the control model are identical. (PDF) [file pone.0159478.s013.pdf]

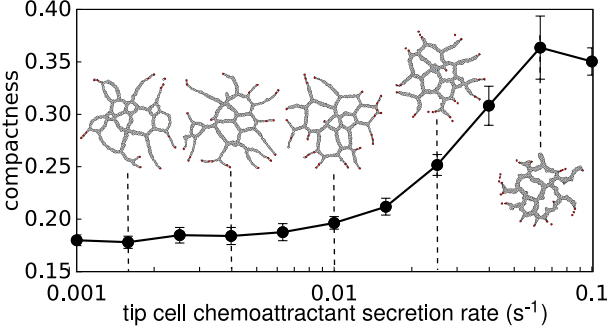

Supplement: S6 Fig — Compactness of the final network (10 000 MCS) with the morphologies for for tip cell Apelin secretion rates of α(tip) = 1.6 ⋅ 10−3, α(tip) = 4.0 ⋅ 10−3, α(tip) = 1 ⋅ 10−2, α(tip) = 2.5 ⋅ 10−2, and α(tip) = 6.3 ⋅ 10−2 as insets. To enable network formation without tip cell chemotaxis J(tip, tip), J(stalk, stalk) and J(tip, stalk) were reduced to 25. Data points show average values for n = 50 simulations with error bars giving the standard deviation. (PDF) [file pone.0159478.s014.pdf]
