# Supplementary material for: Computational Screening of Tip and Stalk Cell Behavior Proposes a Role for Apelin Signaling in Sprout Progression
Source: PLoS One. 2016 Nov 9;11(11):e0159478. doi: 10.1371/journal.pone.0159478 (PMC5102492; doi:10.1371/journal.pone.0159478)
Supplement: S7 Fig — The boxes show the first to third quartile of the data. The whiskers show Q1-1.5 ⋅ IQR to Q3 + 1.5 ⋅ IQR, with IQR = Q3-Q1 = interquartile range, or the most extreme observations if those fall within the range of the whisker. Superimposed on the box plots are the data points. Note that the experiments are done with different collagen gels: Purecol collagen (A,E), Nutacon collagen (B, F, G), and Cultrex rat collagen (C, D, H, I). (PDF) [file pone.0159478.s015.pdf]

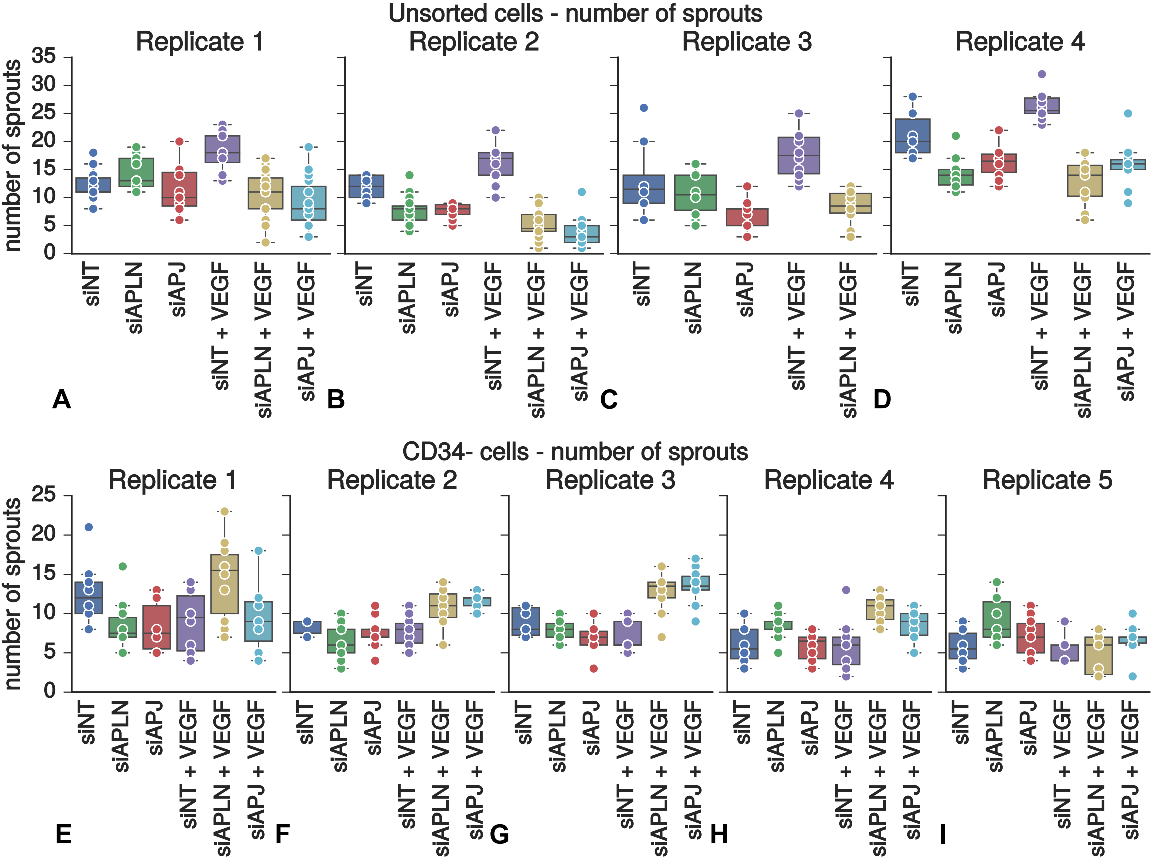

Unsorted cells - number of sprouts
Replicate 1
Replicate 2
Replicate 3
Replicate 4
number of sprouts
siNT
siAPLN
siAPJ
siNT + VEGF
siAPLN + VEGF
siAPJ + VEGF
A
B
C
D
CD34- cells - number of sprouts
Replicate 1
Replicate 2
Replicate 3
Replicate 4
Replicate 5
E
F
G
H
I
